# Using systems thinking and causal loop diagrams to identify cascading climate change impacts on bioenergy supply systems

**DOI:** 10.1007/s11027-021-09967-0

**Published:** 2021-08-18

**Authors:** Fanny Groundstroem, Sirkku Juhola

**Affiliations:** Ecosystems and Environment Research Programme, Faculty of Biological and Environmental Sciences, University of Helsinki, Viikinkaari 1, P.O. Box 65, 00014 Helsinki, Finland

**Keywords:** Bioeconomy, Energy transition, Cascading risks, Indirect impacts, Cross-border impacts

## Abstract

Increased use of bioenergy, driven by ambitious climate and energy policies, has led to an upsurge in international bioenergy trade. Simultaneously, it is evident that every node of the bioenergy supply chain, from cultivation of energy crops to production of electricity and heat, is vulnerable to climate change impacts. However, climate change assessments of bioenergy supply chains neither account for the global nature of the bioenergy market, nor the complexity and dynamic interconnectivity between and within different sub-systems in which the bioenergy supply chain is embedded, thereby neglecting potential compounding and cascading impacts of climate change. In this paper, systems thinking is utilised to develop an analytical framework to address this gap, and aided by causal loop diagrams, cascading impacts of climate change are identified for a case study concerning imports of wood pellets from the United States to the European Union. The findings illustrate how the complexity and interconnectivity of the wood pellet supply system predispose the supply chain to various cascading climate change impacts stemming from environmental, social, political and economic domains, and highlight the value of using system-based analysis tools for studying such complex and dynamic systems.

## Introduction

Modern use of bioenergy (for electricity, heating and transportation) has seen a remarkable expansion in the last decade, mainly because of climate change and energy security concerns (Hoefnagels et al. 2014). Bioenergy has traditionally been used locally, and political efforts to increase its use mainly rely on the presumption that it will be domestically sourced, and hence increase energy self-sufficiency (Mandley et al. 2020). However, international trade in bioenergy products has increased rapidly during the past decade, from around 785 petajoule (PJ) in 2004 to 1250 PJ in 2015 (Junginger et al. 2019). Additionally, there are countries such as Finland that become net importers of bioenergy, if trade in biomass that indirectly ends up in energy production is accounted for (Heinimö 2008). Furthermore, a majority of bioenergy demand or supply scenarios project a further increase in international trade in the future (Daioglou et al. 2020; Mandley et al. 2020). This is oftentimes due to more favourable market conditions (mainly lower prices) for imports from major production regions than domestic production (Lauri et al. 2014; Matzenberger et al. 2015; Rytter et al. 2016), and the fact that the actual market potential for domestic biomass supply is substantially lower than the theoretical potential in many countries, after accounting for environmental, social, technical and economic restraints (Egnell and Börjesson 2012).

Significant uncertainties exist as to how the global bioenergy market will evolve amidst future climate, socioeconomic and technological change, and how an increase in global bioenergy demand will be realised (Kranzl et al. 2014). Demand projections for bioenergy under different climate change mitigation scenarios (see e.g. Bauer et al. 2020; Daioglou et al. 2019), as well as estimations of global and regional bioenergy production potentials (see e.g. Hamelin et al. 2019; Searle and Malins 2015), are readily available, although results vary widely between studies (Mandley et al. 2020). Conversely, studies of future international bioenergy trade are scarce and bioenergy scenarios and models rarely try to map out international trade flows of biomass (Daioglou et al. 2020; Kranzl et al. 2014).

Climate change impact, vulnerability and risk assessments concerning bioenergy have hitherto been isolated to specific segments or aspects (such as demand projections, or economic drivers) (Cronin et al. 2018; Emodi et al. 2019; Schaeffer et al. 2012), specific supply chain nodes (mostly feedstock production) (see e.g. Haberl et al. 2011; Nguyen and Tenhunen 2013; Preston et al. 2017), specific climate change impacts (such as floods or droughts) or confined within national borders (Langholtz et al. 2014). However, the need to understand cascading climate change impacts, defined here as impacts that flow through a network of interconnected system components, affecting the components in different ways (Helbing 2013), has gathered prominence in recent years. Cascading climate change impacts emerge from interdependencies between coupled natural and socio-economic systems in response to changes and feedbacks (Lawrence et al. 2020). The cascading of impacts may be contained locally or span vast geographical areas.

The aim of this paper is to apply systems thinking in order to advance the identification and assessment of potential cascading climate change impacts (hereafter referred to as cascading impacts) that may affect complex international bioenergy supply systems in the future. This is achieved through the creation of an analytical framework for identifying the network structure in which the international bioenergy supply chain is embedded. We apply the framework within a case study and ask the following research question: In what ways could cascading impacts affect imports of wood pellets from the United States (US) to the European Union (EU)? We identify and visualise cascading impacts on the wood pellet supply system through a literature review and causal loop diagrams (CLDs). This type of an approach accounts for the complexity and interconnectivity between and within different systems by identifying relations and connections that have previously been considered in isolation.

## Theoretical background and analytical framework

### Complexity of energy systems and climate change impacts

The energy sector, with its regional and global networks of infrastructure and supply chains, can be thought of as an inherently complex and dynamic system-of-systems, consisting of ‘multiple, heterogeneous, distributed systems embedded in networks at multiple levels that evolve over time’ (Agusdinata and DeLaurentis 2008). A system is defined here as a set of multiple interdependent components, connected through causal relationships, which together express a structured function or purpose. A system can only be fully understood by observing it as a whole i.e. by examining all the interactions between the different components and observing the subsequent performance. A system can be, and often is, composed of several smaller sub-systems, which are connected through networks that allow for movement or communication between them (Haraldsson 2004).

The complexity of energy systems stems from the heterogeneity of and dynamic interdependence between the components of the sub-systems and the complexity of the networks that connect them, as well as the uncertainty related to its future state (Agusdinata and DeLaurentis 2008). The components of the sub-systems consist of various operations and actors that are shaped by different policies, regulatory frameworks and institutions, market rules and regulations (Hoggett 2014), as well as the natural and social environment (Parish et al. 2018). The uncertainty regarding the future state of the system arises from the difficulty in projecting how the different components and the network connections will be affected by climatic, demographic, social and behavioural changes, in addition to technological innovations and emerging products.

Within complex and interconnected systems, individual climate change impacts and risks tend to cascade through the networks, affecting components and actors both geographically and temporally distant from the original impact (Challinor et al. 2018; Groundstroem and Juhola 2018; Hochrainer-Stigler et al. 2020). Such cross-border, systemic, cascading impacts^1^ are further amplified or diminished by social, institutional, political and behavioural factors that affect the perception of the impact, the subsequent responses and the vulnerability and resilience of the system (Challinor et al. 2018). For instance, Shughrue and Seto (2018) show how cascading impacts stemming from natural hazards are readily transferred between urban areas connected by international supply networks, while Bierkandt et al. (2014) and Otto et al. (2017) convey how perturbations may cascade along a supply chain and result in supply disruptions far from the original site of damage. Similarly, Bollinger et al. (2014) highlight the risk of climate change impacts cascading along interconnected infrastructure networks, and the subsequent need for proper management and adaptation responses.

Climate change is a wicked problem (Adger et al. 2018), and social dynamics and complex transmission pathways of cascading impacts are oftentimes not readily quantifiable (Challinor et al. 2018). Hence, it is often advisable to involve a qualitative approach in cascading impact assessments, in addition to or as a basis for quantitative methods, in order to ensure incorporation of all the components, both quantifiable and non-quantifiable, of the system under study (Agusdinata and DeLaurentis 2008; Challinor et al. 2018; Chappin and van der Lei 2014). Systems thinking can be a useful tool for this purpose. Systems thinking was developed to identify and explain relations and interconnections between seemingly unrelated components within complex and dynamic systems. By thinking holistically, a model representation of the system and all its networks is produced, which can further be analysed with e.g. CLDs to highlight components and feedbacks within the system (Haraldsson 2004).

### Analytical framework

**Fig. 1:**
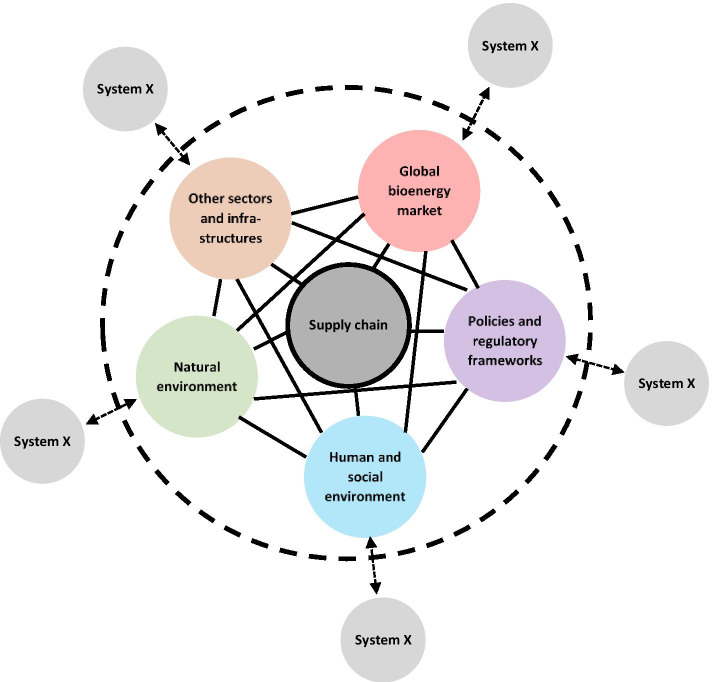
The analytical framework describing the bioenergy supply system. The core supply chain system (dark grey circle in the middle) is connected to other sub-systems (coloured circles) through a complex network (solid black lines). The boundary of the system (dashed black circle) excludes such sub-systems (light grey circles) that do not directly affect the supply chain

Utilising a systems thinking approach, a general analytical framework was developed that lays out the sub-systems and network interconnections of a bioenergy supply system. The core system under study is the supply chain, consisting of cultivation of biomass; harvesting or collection of feedstock; different stages of refining, processing and production; queuing and storage at various stages; transportation, transmission or distribution between different nodes; and end use as e.g. vehicle fuel or heating (An et al. 2011; Awudu and Zhang 2012; Hoefnagels et al. 2014). The operations of the supply chain are directly affected by different actors and factors stemming from the sub-systems of other sectors and infrastructures, the global bioenergy market, policies and regulatory frameworks, the human and social environment and the natural environment. Additionally, the supply chain is indirectly affected through the movement of actors and factors within the complex network that the system is embedded in (Fig. 1).

For instance, the supply chain system might be connected to the water sector if cultivation of certain bioenergy crops require irrigation. Similarly, different stages of refining or processing are dependent on an uninterrupted supply of electricity, while logistics tend to rely on the ICT (information and communication technology) sector for communication and management. Furthermore, competition for land-use and natural resources can create conflicts with other sectors as shown by studies on the water-energy-food nexus (Zhang et al. 2018).

The supply chain is also affected by the rules, regulations and technological innovations of the international bioenergy market. The bioenergy market is essentially defined by the status of and alterations to demand and supply, both of which have been increasing rapidly in recent years. At the same time, the geographically separated centres for demand and supply have resulted in the market becoming increasingly international, a phenomenon that is projected to intensify in the future, both for direct trade in bioenergy products, as well as the significant amounts of traded biomass products that indirectly ends up in energy production (Heinimö and Junginger 2009; Proskurina et al. 2017). Whether a region becomes a competitive player on the global bioenergy market is largely defined by economic factors such as the cost of feedstock production, transport and labour (Lamers et al. 2012), as well as e.g. free trade agreements, anti-dumping and phytosanitary regulations, sustainability requirements, and to a lesser extent country-specific tariffs (considering most countries are members and abide by the rules of the World Trade Organization (WTO)) (Daioglou et al. 2020). Technological innovations, such as cheaper production processes and new energy products, may restructure the global bioenergy market and affect the supply chain operations in different ways.

The supply chain operations, as well as other sectors and the global bioenergy market, are governed by international and regional policies and regulatory frameworks. Renewable energy projects, including bioenergy, are often supported by governments in the form of subsidies, grants, feed-in tariffs or quota systems. In addition, governments may impose environmental taxes or more stringent reporting and sustainability requirements for companies. The aim of policy support is to promote positive externalities, such as energy security, sustainability, climate change mitigation, economic growth, social welfare or job creation, and to spur innovation and maturation of nascent technologies (Lamers et al. 2011; White et al. 2013). Policies affect both the behaviour of individuals and households i.e. the demand side, as well as the decisions and actions of companies i.e. the supply side. While policies, when designed and implemented correctly, tend to be beneficial for renewable energy deployment, inconsistent and poorly designed policies have the opposite effect, hindering the expansion and utilisation of renewable energy. For instance, many renewable energy projects require substantial amounts of credit, which is obtained through loans or investments. If the project is dependent on unstable government support policies, such as a feed-in tariff that is up for re-evaluation in a few years, banks and investors will be reluctant to provide capital to a reasonable price, and the project will most likely be scrapped. In contrast, policies that are set for a 10 or 20-year period and have thoroughly investigated and planned for factors such as social acceptability, uptake rates and the need for infrastructure upgrades, are much more likely to succeed in promoting the deployment of renewable energy projects. In fact, consistent and long-term support policies can be regarded as the most important factor in achieving renewable energy targets (White et al. 2013). Strong supporting policies in both the EU and the US is also the main reason for the significant expansion of global bioenergy production and international trade in biomass in the past decades, despite these policies being targeted at domestic production and consumption (Lamers et al. 2011).

Social and human factors come into play through changes in attitudes, behaviours and perceptions of the various actors operating throughout the system, such as land owners, loggers and farmers, mill and refinery owners, port operators, truck drivers, investors, forestry and farmers’ associations, non-governmental organisations (NGOs) and citizens (Parish et al. 2018). For instance, for policies to go through, a reasonable level of public support is usually required (White et al. 2013). The sustainability of bioenergy is a contested issue, and citizens’ perceptions of bioenergy in general (e.g. regarding the impact on biodiversity, carbon neutrality, competition with other land use), and of specific bioenergy projects (aesthetics, odour, noise, economic and social benefits), can change rapidly (Gold 2011).

Additionally, the bioenergy supply system is embedded in and affected by the natural environment. Agricultural crops or trees used as feedstock for bioenergy are inherently affected by e.g. soil properties, biodiversity and groundwater levels. Changes to species distributions and competitions, among both plants and animals, have far-reaching implications for whole ecosystems and affect the growing conditions for bioenergy feedstock. Moreover, weather and climate affect, and are affected by, the cultivation and management of bioenergy feedstock. Crops and trees are intricately linked to the global carbon cycle, through sequestration of carbon in living material, and the subsequent release of carbon to the atmosphere or soil upon combustion or decomposition (Delucchi 2010). Infrastructure and people are also very much dependent on a stable natural environment and affected by weather and climate.

The complex network structure connecting the sub-systems together, predisposes the supply chain to cascading impacts, risks and failures. Studying complex and dynamic system-of-systems is an extremely laborious task, and therefore, a simplification of the network structure, or inclusion of only some of the sub-systems is usually warranted. The case-specific boundaries of the bioenergy supply system under study should thus include at least those sub-systems that directly affect the core supply chain, and the network they are embedded in, but may exclude indirect interconnections to such systems that do not have a direct connection to the supply chain.

## Methodology

### Justification of the case study

The framework was operationalised through a case study, concerning imports of wood pellets to the EU from the southeast region of the US. Overall, bioenergy accounts for ca. 60% of all renewable energy used in the EU, and this share is projected to increase further in the future (Joint Research Center 2019), as bioenergy is promoted as a cost-effective and efficient means by which the common EU greenhouse gas (GHG) reduction target of 40% below 1990 levels and the renewable energy target of 32% of total energy production by 2030 could be met (EC 2018; Sikkema et al. 2021). Additionally, almost all EU member states have national strategies or other relevant policy initiatives in place or under development to promote the use of bioenergy as part of a transition towards a low-carbon economy (EC 2019). As a result, bioenergy imports from outside Europe are projected to increase substantially, with as much as 60% and 76% of EU demand potentially being met by imports in 2030 and 2050, respectively, compared to ca. 4% currently (Mandley et al. 2020).

**Fig. 2:**
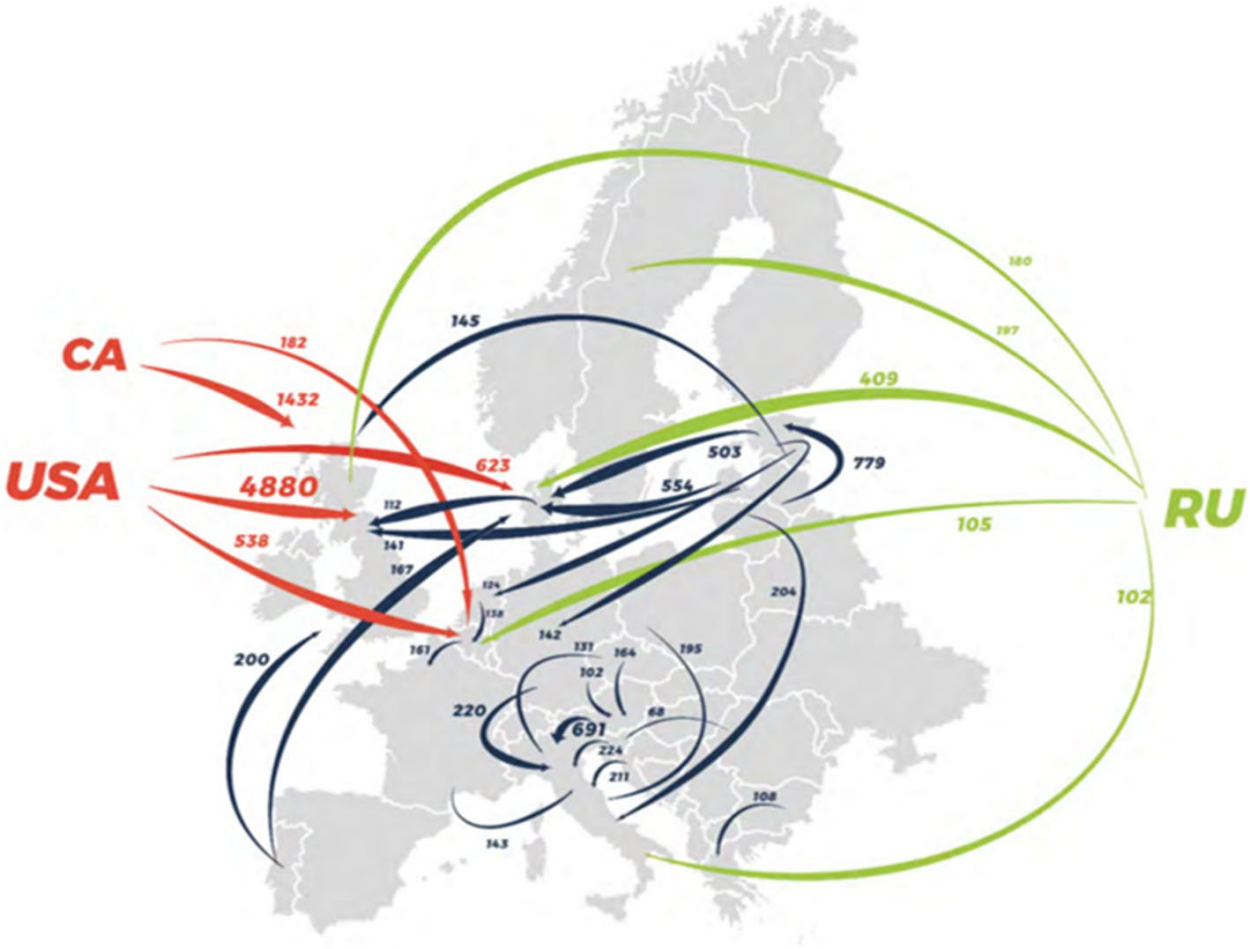
Map of major trade flows of wood pellets to or within Europe in 2018 (in ktonnes) (Calderón et al. 2019)

Wood pellets is the most common form of bioenergy used in the EU, which consumes around half of the global pellet production (amounting to more than 26 million tonnes in 2018), mainly in large scale combined heat and power (CHP) plants. Pellets are a globally traded commodity, with many EU member states, such as the UK, Denmark, Italy and Belgium, importing almost all of their consumed pellets from abroad (Fig. 2) (Calderón et al. 2019). The US accounts for over 60% of imports and 70% of all pellets used for electricity in the EU (Dwivedi et al. 2019; Fingerman et al. 2019), and these shares will likely increase substantially in the future (Johnston and van Kooten 2016; Jonsson and Rinaldi 2017; Sun and Niquidet 2017). During the last ten years, the US pellet production has increased significantly almost exclusively as a response to increasing European demand (Diaz-Chavez et al. 2019) and the US is also the largest exporter of sustainably sourced pellets to Europe (Thrän et al. 2019). Paolotti et al. (2015) showed that importing pellets from the US is in many cases both more economical and more sustainable with regards to GHG emissions, than importing pellets from other European countries. Additionally, the US has vast forest resources, relatively low supply costs, high quality products, and largely complies with European sustainability criteria. The southeast region of the US is the main hub for production and export of pellets, sourcing more than 98% of pellets exported to the EU (Fingerman et al. 2019).

### Methods and data

**Fig. 3:**
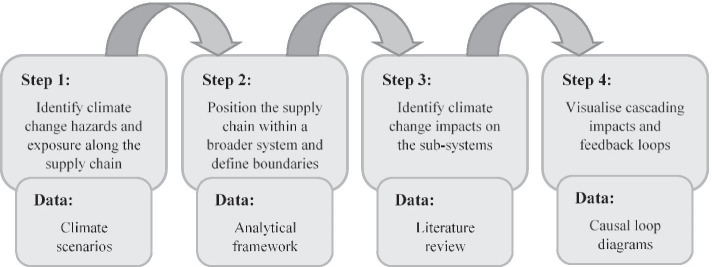
Steps of the analysis and data used

The analysis was carried out in a four-step procedure (Fig. 3). First, direct climate change hazards and exposure in the three main geographical areas in which the wood pellet supply chain is located, namely, the southeast US, North Atlantic Ocean and the port of Rotterdam, were identified based on the US Fourth National Climate Assessment (NCA4), the fifth assessment report of the Intergovernmental Panel on Climate Change (IPCC AR5) and the Royal Netherlands Meteorological Institute’s climate scenarios (KNMI’14), respectively. Second, the bioenergy supply system in which the wood pellet supply chain is embedded was identified based on the analytical framework, and system boundaries were defined. Third, the most prominent climate change impacts on the sub-system components were identified by a literature review. Direct impacts on CHP plants or end use of electricity and heat were omitted from the study as this is highly location specific, whereas the end-user in this study (the EU) is not. Finally, the cascading effect of the impacts and potential feedback loops within the system were visualised in CLDs using a novel layered approach to highlight how complexity in the system emerges through sub-system feedbacks.

The purpose of a CLD is to map out the structure of a system and its networks and reveal causalities and feedbacks within the system (Haraldsson 2004). CLDs are commonly used alongside systems thinking to facilitate understanding and analysis of the system under investigation (Sanches-Pereira and Gómez 2015). A CLD is composed of variables connected by arrows that indicate unidirectional causal links between the variables. An arrow from variable *X* to variable *Y* with a positive sign (+) represents a positive link, indicating that a change in variable *X* produces a change in the same direction in variable *Y*; thus, if *X* increases, *Y* increases. An arrow with a negative sign ( −) represents a negative link, which means that a change in variable *X* results in a change in the opposite direction in variable *Y*; thus, if X increases, Y decreases. Note that the signs only indicate the direction of change (same or opposite) of the affected variable, and have nothing to do with whether the change is increasing or decreasing, beneficial or detrimental. Some of the links between variables are characterised by delays, which may have implications for the whole system. Delays are shown in the CLD by crossing lines on an arrow. One crossing line indicates a short delay, while two crossing lines indicate a long delay. Generally, the longer the delay, the larger the implications for the system (Sanches-Pereira and Gómez 2015). The timeframes for short versus long delays need to be defined for each study separately as this is highly system specific.

**Fig. 4:**
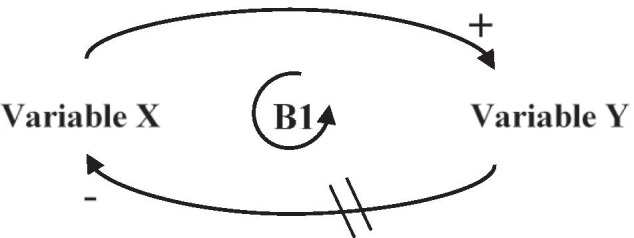
An example of a simple feedback loop, where variable *X* has a positive effect on variable Y (thus inducing a change in variable *Y* in the same direction), which in turn has a negative effect (although delayed) on variable *X* (counteracting the initial effect), resulting in a balancing feedback loop

A CLD offers an opportunity to identify feedback effects in the system, which may point to potential future trajectories of change. Feedback effects arise when variables affect each other in a cascading manner, ultimately leading back to a previous variable, creating a feedback loop (Fig. 4). A feedback loop can be either reinforcing (R), if events or behaviours created by the variables in the loop amplify each other, leading to unbounded growth or decline, or balancing (B), if some variables create counteracting changes, resulting in equilibrium. An easy way of assessing the effect of the feedback loop is to count the number of negative signs in the loop; an even number results in a reinforcing loop, and an odd number results in a balancing loop (Kirkwood 1998).

In this paper, climate change impacts in the CLDs are divided into incremental climate change (i.e. temperature increase and precipitation change), extreme weather (comprising heat waves, droughts, wildfire, heavy rainfall and inland flooding) and sea level rise (including coastal erosion, storm surges and coastal flooding). Additionally, the impact of increasing atmospheric carbon dioxide (CO_2_) on tree growth and productivity is depicted separately. In addition to positive (** +**) and negative ( −) effects, arrows with the sign **o** are present in some of the CLDs, representing effects that can be either positive or negative depending on the situation. Since climate change impacts are inherently characterised by unknown time lags, delays in the CLDs have been sparsely highlighted only for the impacts that are believed to materialise significantly later than the rest.

### System boundaries

As this study is concerned with cascading impacts of climate change, the system boundaries of the wood pellet supply system only include such actors and factors that both influence the supply chain operations, either directly or indirectly through another sub-system, and simultaneously could be affected by climate change.

At the core of the wood pellet supply system under study is the supply chain, which starts with the acquisition of feedstock from pine (and to a lesser extent different hardwood) plantations and forests in the southeast US. After logging and natural drying in the forests, the wood is collected and transported by truck either directly to nearby pellet mills in the case of pulpwood and other low-grade wood, logging- and forestry residues, and thinnings (comprising ca. 60% of the feedstock), or to sawmills or different wood product manufacturers, from where residues and by-products are later acquired by the pellet mills (comprising the remaining 40%) (Fingerman et al. 2019; Goetzl 2015; Hoefnagels et al. 2011). The finished pellets are transported by truck, rail or barges to an international port on the US east coast, from where they are shipped over the North Atlantic Ocean to Europe (mostly to Rotterdam, which is the largest port on the Atlantic coast and the main port for transhipments in Europe) (Diaz-Chavez et al. 2019; Fingerman et al. 2019). From there, the pellets are further transported to CHP plants throughout the EU. Storage takes place at the sawmills, pellet mills, ports and CHP plants.

The sub-system of other sectors and infrastructures is confined to the forestry and wood manufacturing sectors, tourism, nature conservation, agriculture, electricity production and distribution and the ICT sector. The pellet industry collaborates and competes directly with the rest of the forestry and wood manufacturing sectors for access to wood resources (Conrad et al. 2011). In general, sawmilling and other forest industries requiring high-quality wood are collaborators with the pellet industry, who uses residues and by-products from harvest-, thinning- and milling operations of these industries, while the pulp and paper industry is a competitor as it utilises the same feedstock (Johnston and van Kooten 2016; Jonsson and Rinaldi 2017). The tourism sector, recreational use of forests and federal nature conservation compete for access to undisturbed forests, while agriculture competes for land-use rights (Beach et al. 2015). Pellet production is connected to the electricity sector since e.g. drying, pressing, milling and storage require electricity (Hansson and Hackl 2016; Uasuf and Becker 2011), while many operations, such as transport, depend on reliant ICT services (Markolf et al. 2019). In contrast, other sectors such as the water sector, are outside the system boundaries as it does not have a significant direct impact on the supply chain per se, although it is tightly connected to e.g. electricity production and agriculture. Likewise, population growth and urbanisation in the southeast US increase the value of land adjacent to urban areas, and subsequently the likelihood of forests being allocated to urban development (Wu et al. 2014), thus competing for land-use. However, it is not clear whether population growth and urbanisation are affected by climate change, and they are therefore not included in this study.

Price fluctuations of pellets, pellet demand and supply trends, research and innovation in the bioenergy field, as well as quality standards of pellets, are included in the study through the global bioenergy market sub-system. Pellet prices in the EU are heavily dependent on developments on the global market, such as demand and supply trends, currency exchange rates and local events disrupting supply (Parish et al. 2018). The quality and characteristics of the pellets available on the international market may change in the future due to technological innovations, which may e.g. increase energy density or decrease lifecycle GHG emissions (Hansson and Hackl 2016).

Policies and regulatory frameworks included within the system boundaries are national (US), regional (EU) and international climate change and energy policies, as well as sustainability criteria that may affect the pellet supply chain. In fact, national and EU climate and renewable energy goals and targets are the main drivers of the transatlantic pellet trade (Parish et al. 2018), while EU sustainability criteria set out rules for pellet feedstock plantation management.

The boundaries of the sub-system of the human and social environment can be challenging to define, due to the complexity and untraceable nature of human behaviour, thoughts and perceptions. Included within the system boundaries of this study are southeast US forest owners and foresters, employees in the forest- and pellet industries, and both US and EU citizens and NGOs, whose actions can influence and affect the operations of the supply chain. For instance, the availability of feedstock for pellet production is first and foremost in the hands of the private forest owners, whose decision to harvest is affected by personal values, financial needs and life events, and weighted against other uses of the forest, such as recreation and hunting (Butler et al. 2017; Dale et al. 2017). Additionally, the pellet industry is subject to fluctuating support from citizens and NGOs. As the industry is a significant employer in the southeast US, it is generally perceived by citizens as benefitting the community, although the highly export-oriented focus of the industry is sometimes given a negative connotation. However, some US NGOs have raised concerns about biodiversity loss, deforestation and high disturbance rates (Diaz-Chavez et al. 2019), due to a high conversion rate of natural forests to pine plantations in response to increased pellet demand in the southeast US (Duden et al. 2018; Wade et al. 2019).

Finally, the sub-system of the natural environment is the very foundation upon which the wood pellet supply system depends on. Included within the system boundaries are the ecosystems that southeast US forests and plantations are a part of, specifically focusing on the growing conditions of forests, variations in species distributions, the prevalence of insects and pathogens, as well as the ecosystem services that southeast US communities rely upon. The climate of the southeast US is excellent for rapid tree growth and regeneration, which has spurred forestry developments in the region, at the expense of natural forests (Cristan et al. 2016). Much of the southeast US is part of the North American coastal plain, which was designated a biodiversity hotspot in 2015 (Noss 2016). The coastal plain supports a rapidly increasing population, a burgeoning tourism sector, and valuable cultural resources, all of which are exposed to sea-level rise, flooding, coastal erosion, storm surges and saltwater intrusion, with implications for ecosystem resilience, human wellbeing and vulnerability, and infrastructure. Ecological diversity in the region is high and the ecosystems provide a range of societal benefits, such as improved water and air quality and flood protection (Carter et al. 2018).

## Results

### Projected climate change in the supply chain regions

Temperatures in the southeast region of the US are projected to increase by ca. 1.9–2.4 °C by mid-century and by 2.5–4.3 °C by late-century, with the lower end representing RCP4.5 and the higher end RCP8.5.^2^ This is slightly less than the country average, mainly due to increased evapotranspiration, which releases latent heat. Conversely, the southeast is likely to see a higher increase on average in the frequency and intensity of heat waves (a 6-day period with a maximum temperature above the 90^th^ percentile). Additionally, the number of days with a maximum temperature above 32 °C is projected to rise by 40–50 days by mid-century under RCP8.5 (Vose et al. 2017). The change in total seasonal precipitation is likely to remain small compared to the natural variation in the southeast. Generally, increased precipitation is expected in the northern areas of the southeast region, while decreased precipitation is projected for the southern areas (Easterling et al. 2017). However, the severity and frequency of extreme precipitation events are projected to increase substantially even in RCP4.5 (Carter et al. 2018). The frequency and magnitude of agricultural droughts (i.e. conditions of soil moisture deficits) will likely increase in all seasons in the future, due to increased evapotranspiration rates (Wehner et al. 2017).

Changes to fire regimes (i.e. the frequency, pattern, size, intensity, severity and season) are projected to have a profound effect on the southeast US, which already experiences the highest number of wildfires in the country, as well as the largest area burned by prescribed fire. In the future, increased temperatures and prolonged droughts are likely to result in more frequent wildfires and reduce the effectiveness of prescribed fire. Furthermore, rapid urbanisation along the edges of forests reduces the possibility for using prescribed fires (Carter et al. 2018).

Sea levels are projected to rise more than the global average (projected to be as much as 1.5–2.5 m by the end of the twenty-first century) in the southeast US, due to local land sinking and groundwater withdrawal (Carter et al. 2018). Subsequently, coastal flooding and storm surges are projected to occur frequently in the future, potentially daily in some areas (Sweet et al. 2017). Hurricanes are a major concern for the southeast, but future changes to the frequency of hurricanes due to climate change are unclear. However, studies show that the intensity and precipitation rates of hurricanes are likely to increase with warmer temperatures (Easterling et al. 2017).

The already observed sea surface temperature increase in the North Atlantic Ocean is projected to continue, with ca. 0.5–3 °C increase expected during the period 2010–2099, depending on the emission scenario. Sea level is also expected to rise, although this will only be discernible along the coasts. A northward shift in storm tracks, along with increased storm activity and resulting surface wave heights, have been observed in the North Atlantic, but it is unclear how these trends will evolve in the future, due to a gap in research (Hoegh-Guldberg et al. 2014; IPCC 2014).

The coastal area of the Netherlands is projected to experience less pronounced warming than the eastern and southern areas of the country, although temperatures in the Netherlands are projected to increase slightly more than the global average. Conversely, scenarios suggest that the coastal area may experience greater winter precipitation increases than the rest of the country. Summers are expected to become slightly drier in the future. Sea levels are projected to rise 15–40 cm by 2050, and 25–80 cm by 2085. By 2100, the rise may be as high as 100 cm. Due to the already low-lying area, storm surges, floods and coastal erosion will likely become major challenges for the coastal region of the Netherlands in the future (KNMI 2015).

### Cascading climate change impacts on the wood pellet supply system

#### The core supply chain

Tree growth and productivity are directly affected by changes to the climate, especially increasing temperatures and altered precipitation patterns. For instance, high temperatures and droughts are major causes of tree damage and mortality. Wildfires are a major threat to both natural forests and plantations in the southeast US and will require improved management strategies in the future (Carter et al. 2018). Increased numbers of damaged or dead trees can temporarily increase the supply of feedstock to pellet mills but will decimate the supply in the long run (Barrette et al. 2015). In contrast, increasing atmospheric CO_2_ levels are generally projected to benefit forests globally, increasing the rate of photosynthesis and tree growth. However, the magnitude of the CO_2_ fertilisation effect is unclear, and tree growth would still be inhibited by lack of other nutrients, and by droughts in the southeast US (Susaeta et al. 2014).

Harvesting wood may be rendered impossible due to extreme weather events, such as heavy storms, extreme rainfall, flooding or wildfires, as e.g. operating of heavy machinery may become difficult or impossible. Wildfires and heavy rainfall also impact the natural drying of timber on the forest floor. Wildfires are also a risk to pellet mills or other forestry and storage facilities located close to forests. Facilities could also be susceptible to e.g. flooding, hurricanes or sea level rise, depending on their geographic location (Acuna and Strandgard 2017).

Self-heating and ignition of pellets, as well as biological degradation and pest infestation, are common problems during storage, with the risk increasing with longer storage periods. Dust explosions are dangerous incidents that can happen during any stage of the handling process, triggered by electrostatic discharge, hot surfaces or high friction temperatures (Dafnomilis et al. 2018; Kymäläinen et al. 2015). All these risks may be intensified by a warmer and wetter climate. Pellet storage facilities need to be enclosed to prevent moisture build-up and spreading of dust, and they need to be large enough to accommodate large quantities of pellets while ensuring adequate ventilation (Dafnomilis et al. 2018; Whittaker and Shield 2017), qualities that many storage facilities in the southeast US lack (Diaz-Chavez et al. 2019). Many ports have storage facilities for pellets right next to the quay, in order to minimize handling of the pellets (which may cause damage), and reduce the time spent outdoors. This leaves the storage facilities vulnerable to storm surges and sea level rise. During rain, loading and unloading of pellets is usually seized to prevent degradation of the pellets (Dafnomilis et al. 2018). Increased precipitation may thus cause delays in supply or extend the time of storage at ports, hence increasing the risk of fire and biological hazards.

Disruptions to the transportation network, including ports, have been recognised as a significant risk to the whole economy of a region. Extreme weather events may cause detours, delays, accidents or cancellation of transport, incurring substantial costs for various actors along the supply chain (Becker et al. 2018; Jaroszweski et al. 2010; Koetse and Rietveld 2009). During 2014–2016, four major inland flood events occurred in the southeast US, causing casualties, injuries and health problems, as well as billion-dollar damages to property and infrastructure. Additionally, coastal property and transportation infrastructure in the southeast US are very vulnerable to sea level rise, hurricanes, storm surges and flooding. Many southeast US cities are projected to experience more than 30 days of high tide flooding annually by 2050 even in a low-emission scenario; the port cities of Savannah and Wilmington already experienced all-time records of 38 and 90 days, respectively, of coastal flooding in 2016 and 2015 (Carter et al. 2018). Bridges, roads and rail networks in the region are projected to experience the largest damages in the country by mid-century under both low and high emission scenarios (Carter et al. 2018; EPA 2017). Additionally, high winds may blow vegetation onto roads and rail lines and cause instability of high-sided vehicles. High temperatures can cause thermal loading of roads (resulting in expansion, bleeding or rutting of asphalt), and buckling of rails (Dawson et al. 2016).

The main risks for water transport are mostly evident at ports. Port facilities are susceptible to impacts from sea level rise, strong winds and storm- and tidal surges. For instance, high waves can damage docking structures or berthed ships, or cause coastal erosion or sedimentation along the port channel. Flooding may impede overland access to the port or damage facilities in the area, while strong winds may prevent ships from docking or hinder loading/unloading of cargo (Yang et al. 2018). Heavy storm surges can dislodge cargo containers and damage terminal buildings and equipment (DOE-EPSA 2015). During Hurricane Irma in 2017, several major ports in the southeast US had to be closed for operation for several days (Carter et al. 2018). Increased storminess and wave height along North Atlantic shipping routes could be hazardous to ships and force switching to longer, but less storm prone routes, resulting in increased prices and supply delays (Arent et al. 2014), as well as the need to allocate more resources to ship maintenance or upgrade ship structures (Bitner-Gregersen et al. 2018). Transportation may also experience fuel supply disruptions and price spikes if fuel production and distribution is adversely affected by climate change (DOE-EPSA 2015).

**Fig. 5:**
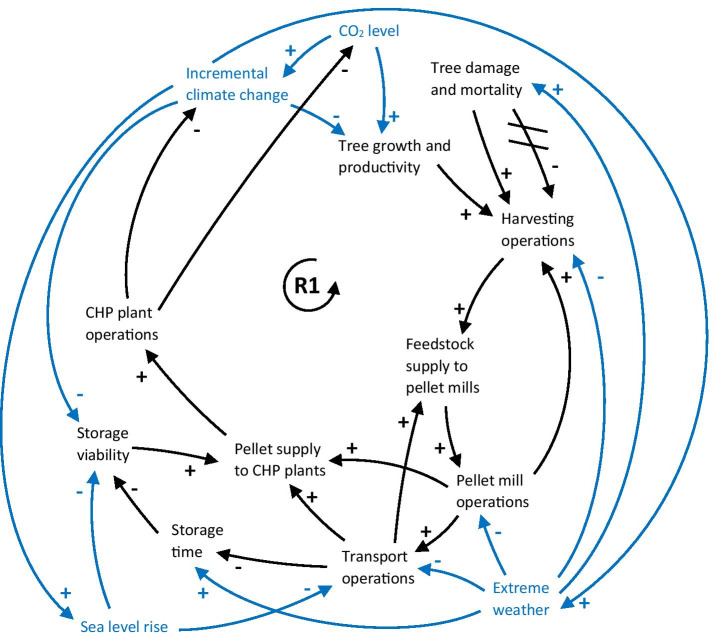
CLD showing the nodes and links of the core supply chain (in black), and the potential for climate change impacts (in blue) to affect the system. R1 refers to the supply chain loop described in the text. A description of every node and link can be found in Table 3 in the Appendix

In summary, every node in the core supply chain is susceptible to climate change impacts that may cascade through the supply chain and disrupt pellet supply to CHP plants, causing a reduction in national bioenergy production (Fig. 5). This may hinder the fulfilment of national emission reduction targets and potentially exacerbate climate change if fossil fuel-based energy production needs to be ramped up. As a hypothetical example, the reinforcing feedback loop R1 (the supply chain loop) shows how increased temperatures and changing precipitation patterns negatively affect tree growth and productivity, leading to decreased opportunities for harvesting operations. Consequently, feedstock supply to pellet mills is reduced, pellet mill operations are shut down, causing pellet supply disruptions at CHP plants, and hence reduced CHP plant operations. As the CHP plants are no longer able to run on pellets, coal is used instead, which further exacerbates climate change. Alternatively, the cyclical nature of the supply chain means that the feedback loop R1 could start with any node that is affected by climate change (such as reduced harvesting operations due to extreme weather events, disruptions to the transport network due to sea level rise, or degradation of pellets during storage due to increased temperatures), which would result in reduced production of pellet-based electricity and heat, a ramp-up of coal-fired power plants and a further increase of climate change.

#### Other sectors and infrastructures

**Fig. 6:**
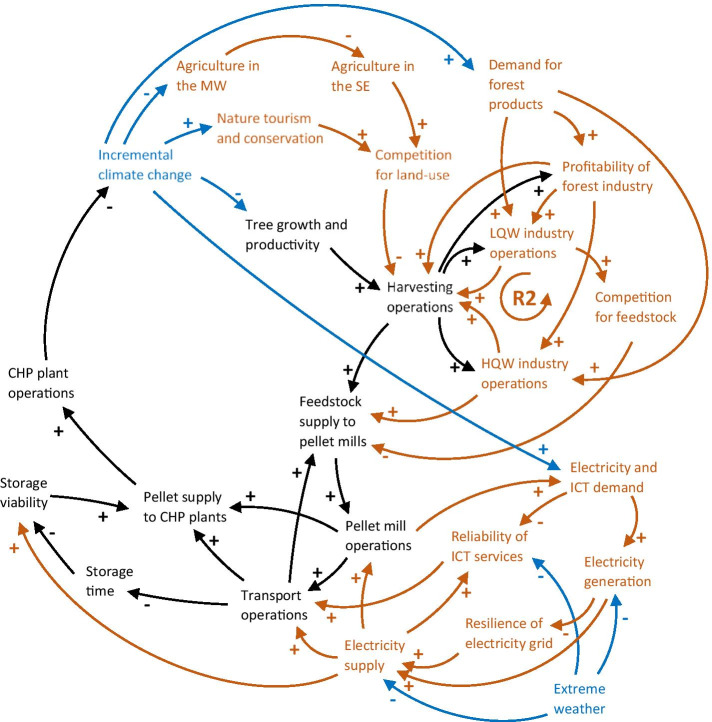
CLD showing how the core supply chain (in black) is connected to other sectors and infrastructure (in light brown). More connections mean greater potential for climate change impacts (in blue) to cascade through the network. R2 refers to the forest industry loop described in the text. Some connections identified in the previous CLD, but not directly related to the new connections, have been removed to improve readability. MW = Midwest US, SE = southeast US, LQW = low-quality wood, HQW = high-quality wood. A description of every node and link can be found in Table 4 in the Appendix

Demand and price projections for timber and other wood products are uncertain but have been trending downwards in the US since the 1990s (Vose et al. 2018). However, an upsurge in future forest product demand has been projected by e.g. Wade et al. (2019), which would result in increased profitability and hence increased operations of the forest industry. This is also reflected in the reinforcing feedback loop R2 (the forest industry loop) in Fig. 6, which shows how increased forestry operations incentivise streamlining and ramping up of harvesting operations, further increasing the profitability of the forest industry. The effect on the pellet industry is twofold: if high-quality wood industry operations are increased, the availability of residues for pellet production also increases, while the opposite is true when low-quality wood industry operations are increased, due to increased competition for feedstock (Lal et al. 2011; Susaeta et al. 2014).

The profitability of the forest industry could decrease if adverse climate change impacts on forests and plantations, such as wildfires or droughts, affect the availability of wood, which would also reduce the supply of residues to pellet mills. Simultaneously, decreased competition from other low-quality wood operations due to reduced profitability could temporarily redirect the remaining residues to pellet mills (Lal et al. 2011). Additionally, increased tree growth due to CO_2_ fertilisation could potentially result in increased feedstock production in the long run and hence lower prices and increased demand for forest products. However, recent hurricanes in the southeast US have previously resulted in billions of dollars in economic losses for the forest industry, which suggests that economic losses are likely to outweigh any positive gains (Susaeta et al. 2014).

Several studies project that recreational visits to forests will increase in a warmer climate, as forests provide shade and lower temperatures than urban areas (Lal et al. 2011). This may entail greater pressure from the public and tourism sector to keep forests intact. Additionally, the need for nature conservation may increase if threats to forest biodiversity and wildlife are anticipated due to climate change. However, as the southeast US forests are mainly privately owned and not a hotspot for nature tourism, the effect of these pressures on land-use patterns is uncertain. Furthermore, concerns over reduced suitability of agricultural land in the Midwest US due to climate change have spurred an interest in expanding agricultural areas in the southeast (McNulty et al. 2015), which may prompt some southeast forest owners to shift to agriculture in the hope of greater revenues. All these factors affect harvesting operations, and hence the pellet industry, as competition for land-use increases.

Climate change induced disruptions to electricity and ICT services could have cascading risks for all nodes in the supply chain (Horrocks et al. 2010). For instance, electricity outages could inhibit operations at mills or ports (Uasuf and Becker 2011), while making road transportation more hazardous by shutting down road lighting and traffic signals. Fant et al. (2020) estimated future climate change induced damages, and subsequent economic costs, to electricity transmission and distribution infrastructure in the US to be substantial, with the southeast region being one of the most vulnerable areas. Furthermore, the dependence on and demand for electricity and ICT services will most likely accentuate in the future due to e.g. population growth, the electrification of transport and the introduction of smart grids and ‘internet of things’ (Markolf et al. 2019; Steinberg et al. 2020), potentially leading to price increases or electricity grid overloads (Martinich and Crimmins 2019). Increased demand could also lead to improvements in and build-outs of the electricity and ICT supply networks, thus benefiting supply chain operations.

#### Global bioenergy market

**Fig. 7:**
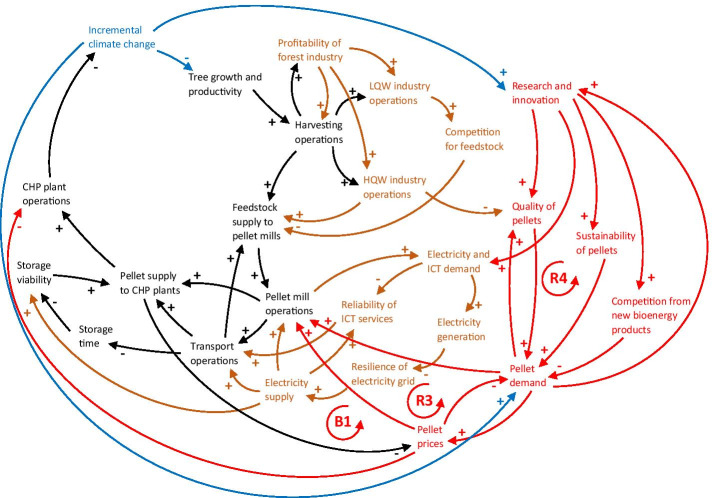
CLD showing how the sub-systems of the core supply chain (in black) and other sectors and infrastructure (in light brown) are connected to the global bioenergy market (in red). More connections mean greater potential for climate change impacts (in blue) to cascade through the network. R3 refers to the pellet demand loop, B1 to the price loop and R4 to the innovation loop described in the text. Some connections identified in the previous CLDs, but not directly related to the new connections, have been removed to improve readability. LQW = low-quality wood, HQW = high-quality wood. A description of every node and link can be found in Table 5 in the Appendix

Climate change may affect the global bioenergy market, and through both direct and indirect connections, these effects may cascade through the network and impact the supply chain (Fig. 7). For instance, adverse climate change impacts on feedstock production may increase the price for pellets, which in turn increases supply costs for CHP plants and subsequently affects the price paid by consumers (Langholtz et al. 2014). Furthermore, the pellet market is characterised by long-term contracts with suppliers sourcing from specific plantations (Roni et al. 2018), which increases the risk of supply shortages due to local disruptions induced by climate change. In the southeast US, pellet demand and production are expected to increase (Wade et al. 2019), which may redirect higher quality pulpwood and saw logs directly towards pellet production, resulting in higher quality pellets. On the other hand, a simultaneous increase in demand for high-quality wood products would leave mainly low-quality wood and by-products for pellet production purposes (Diaz-Chavez et al. 2019). This may complicate the fulfilment of specific industrial-grade quality standards of pellets used in CHP plants in the EU (Olsson and Hillring 2014), perhaps dampening demand. The counteracting forces of demand and price fluctuations are illustrated in Fig. 7 by two simplified feedback loops. The reinforcing pellet demand loop (R3) shows how increasing pellet demand enhances pellet mill operations and causes pellet supply to CHP plants to increase, resulting in a pellet price drop, which further increases demand. The balancing pellet price loop (B1) shows how low pellet prices negatively affect the profitability of pellet mill operations, thus reducing pellet production and supply to CHP plants, causing pellet prices to increase once again in response to a pellet shortage on the market.

Another set of counteracting forces are found when looking at the effects of increased research and innovation, brought about by the need for more climate-friendly products, on pellet demand. On the one hand, research and innovation may result in pellets of higher quality and more sustainable production processes, which would increase the demand for pellets and further incentivise research and innovation in the field of pellet production (Mandley et al. 2020), as depicted by the reinforcing innovation loop R4 in Fig. 7. For instance, innovations such as bioenergy with carbon capture and storage (BECCS) could provide solutions to the ‘carbon debt’ resulting from biomass combustion^3^ and increase the sustainability of bioenergy, resulting in increasing demand, provided that social and political barriers are overcome. Although the uptake and commercialisation of BECCS is yet to be realised at scale, most mitigation scenarios that limit the global temperature rise to 1.5–2 °C assume substantial utilisation of BECCS (Fridahl and Lehtveer 2018).

On the other hand, pellet-based energy production will likely face competition from new types of bioenergy in the future, such as pyrolysis oil, as well as from other uses of lignocellulosic biomass in accordance with national bioeconomy transition strategies, thus dampening the demand for pellets. However, projections regarding the uptake and commercialisation of new bioenergy forms or biobased products are ambiguous. Furthermore, emphasising the ‘cascading principle’ for biomass utilisation could result in more bioenergy being produced from used products at the end of their lifetime (Mandley et al. 2020) and lessen the demand for pellets made from primary sources.

#### Policies and regulatory frameworks

**Fig. 8:**
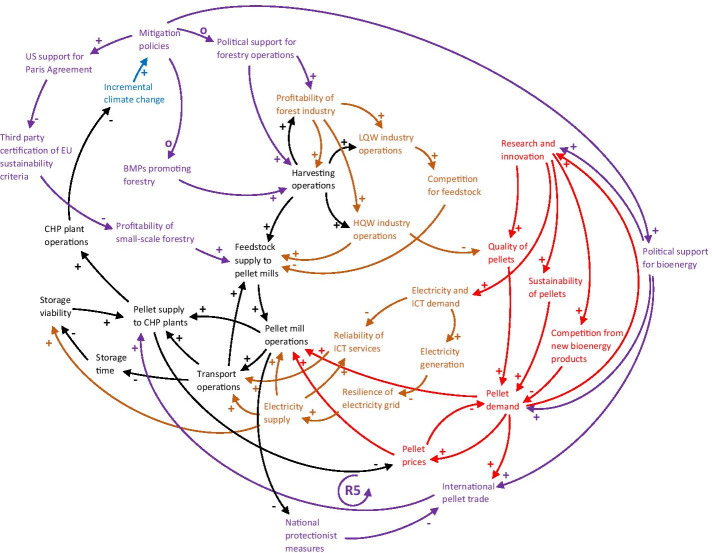
CLD showing how the sub-systems of the core supply chain (in black), other sectors and infrastructure (in light brown) and the global bioenergy market (in red) are connected to policies and regulatory frameworks (in purple). More connections mean greater potential for climate change impacts (in blue) to cascade through the network. R5 refers to the protectionist loop described in the text. Some connections identified in the previous CLDs, but not directly related to the new connections, have been removed to improve readability. LQW = low-quality wood, HQW = high-quality wood, BMPs = best management practices. A description of every node and link can be found in Table 6 in the Appendix

Both the supply chain operations themselves, as well as the global bioenergy market, are heavily influenced and guided by national, regional and international climate change mitigation policies (Fig. 8). Political support for bioenergy is likely to increase as climate change progresses (Mandley et al. 2020), resulting in a steady increase in pellet demand in the EU in the future. However, whether managed tree plantations sequester more or less carbon than natural forests is an ongoing debate that may shift political support from promoting forestry operations to reverting forests to a more natural state to mitigate climate change (Webster 2019), influencing the profitability of the forest industry and its operations. Adverse impacts on forests may also result in protectionist measures being implemented by national governments, as has occurred in the food sector: Local disruptions to agricultural yields have resulted in political decisions to ban exports of the affected commodity, causing a shortage on the global market and price spikes around the world (Challinor et al. 2018). A similar scenario could play out for bioenergy feedstock, as illustrated in Fig. 8: the reinforcing protectionist loop (R5) highlights how adverse climate change impacts on pellet feedstock and hence pellet production in US pellet mills may result in national protectionist measures, which would hamper the international pellet trade, reducing pellet supply to European CHP plants, incurring a pellet price spike, resulting in reduced pellet demand, which would hamper pellet production globally.

The EU RED II (Renewable Energy Directive) specifies sustainability criteria for forest biomass used for large scale (> 20 megawatt) production of fuels, electricity or heating and cooling, to ensure legality of harvesting, forest regeneration, protection of high conservation value areas, minimisation of impacts on soils and biodiversity and maintaining the long-term production capacity. Compliance can be demonstrated through national or regional legislation comprising the harvesting area, or through internationally recognised third party legislation at the forest holding level. Additionally, criteria pertaining to minimising negative effects on the forest carbon stock can be verified by assuring that the country of origin is a party to the Paris Agreement, has a national system for LULUCF (land-use, land-use change and forestry) reporting, or that appropriate management systems are in place at the forest holding level (EC 2018). Dwindling US support for the Paris Agreement as was seen during the President Trump era, and a subsequent withdrawal, would require US pellet producers to show compliance with EU sustainability criteria through e.g. third-party certification or private standards (Webster 2019). Many large forest owners and pellet manufacturers have voluntarily joined a certification program, but this is often prohibitively expensive and difficult for small private landowners (Diaz-Chavez et al. 2019). In fact, according to Poudyal et al. (2019), green certification among southeast US private forest owners is rare.

Approximately 82% of the forests in the southeast US is privately owned (Oswalt et al. 2018), and hence, the US government has no authority to require any form of certification of sustainability. However, various state or federal entities (e.g. US Forest Services, or US Environmental Protection Agency) give recommendations for land-use and best management practices (BMPs) to private forest owners, in order to ensure sustainable use and compliance with federal nature conservation laws. If new evidence of climate risks to, or negative effects from bioenergy utilisation on forests emerge, recommendations for BMPs for private forest owners may change (Parish et al. 2018), affecting the decision by forest owners to allow logging and other forestry practices in their forests. However, incorporating climate change projections into management recommendations has hitherto been scarce (Carlton et al. 2014).

#### Human and social environment

Several factors influence how private forests are being used in the US. In general, the larger the forest holding, the more inclined are the forest owners to engage in harvesting. A fair price, a steady market and stimulating investment opportunities, but also prospects to address environmental problems or climate change and benefit the local community, have been found to influence the decision by private forest owners to sell wood specifically for bioenergy production (Paula et al. 2011). A strong (weak) timber market means more (less) incentives for private forest owners to increase the forested area and implement forest adaptation management measures that increase resilience to climate change (Duden et al. 2017), such as diversifying the forest to include different species of different age, changing to more tolerant tree species, reducing tree density, using prescribed fire, protecting watersheds or adapting forest roads to flood hazards (Susaeta et al. 2014; Vose et al. 2018). An increased availability of harvesting and thinning residues from enhanced adaptation management operations may increase the availability of feedstock to pellet mills, but simultaneously shift the feedstock supply towards more low-quality wood (Fingerman et al. 2019).

Boby et al. (2016) surveyed climate change perceptions among professional foresters working for private forest owners in the southern US and found that 60% believe that climate change is real, but only ca. 14% attributed climate change solely to human causes. Personal observations of climate change impacts are strongly related to the perception of climate change as a real threat (Boby et al. 2016), and scepticism towards climate change hinders the implementation of adaptation measures (Morris et al. 2016). Private forest owners who believe in human-caused climate change tend to be more inclined to not engage in harvesting and instead promote the regeneration of natural forests. A perceived increase in climate change impacts or heightened awareness of climate change among private forest owners and foresters may therefore reduce motivation for harvesting, while simultaneously incentivise adaptation management (Khanal et al. 2017). However, increased awareness of climate change may influence the decision of forest owners to sell feedstock specifically to pellet mills in either direction, depending on whether they perceive bioenergy production to be a viable mitigation strategy or not (Dulys-Nusbaum et al. 2019; Gruchy et al. 2012).

The forestry sector is associated with rural areas, where the population tends to be disproportionately more vulnerable to climate change than in urban regions, due to poverty, high unemployment rates, limited access to healthcare and an aging population. In fact, the southeast US is struggling with persistent poverty in many counties (Lal et al. 2011) and a range of socially and economically adverse impacts on different sectors is projected, making the region one of the most vulnerable in the country (Hsiang et al. 2017). Poverty substantially reduces the adaptive capacity of the communities. For instance, Gaither et al. (2011) showed that socially vulnerable people in the southeast US are less likely to implement wildfire prevention measures on their land, and less able to recover from wildfires than more affluent people in the region.

**Fig. 9:**
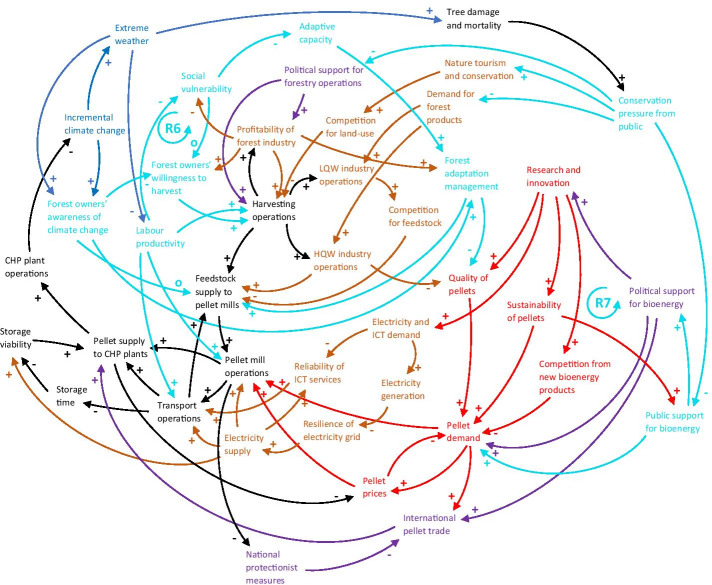
CLD showing how the sub-systems of the core supply chain (in black), other sectors and infrastructure (in light brown), the global bioenergy market (in red) and policies and regulatory frameworks (in purple) are connected to the human and social environment (in turquoise). More connections mean greater potential for climate change impacts (in blue) to cascade through the network. R6 refers to the social vulnerability loop and R7 to the public support loop described in the text. Some connections identified in the previous CLDs, but not directly related to the new connections, have been removed to improve readability. LQW = low-quality wood, HQW = high-quality wood. A description of every node and link can be found in Table 7 in the Appendix

Additionally, labour productivity is expected to decrease substantially due to extreme heat; by the end of the century under RCP8.5 without adaptation, the southeast US is projected to experience a loss of 570 million labour hours annually in high-risk sectors, including forestry, compared to a 2003–2007 baseline (EPA 2017). The reinforcing social vulnerability loop (R6) in Fig. 9 shows the connection between the forest industry and social wellbeing: Increased awareness of climate change may decrease some forest owners’ willingness to engage in harvesting operations, which negatively affects the profitability of the forest industry. This in turn increases social vulnerability of the associated community as unemployment rates increase, also negatively affecting the rest of the forest owners in the area, who may be forced to shift to more profitable activities such as agriculture or nature tourism, further reducing the profitability of the forest industry.

Pine plantations are less biodiverse than natural forests (Duden et al. 2018) and hence potentially more vulnerable to climate change induced disturbances, which further increases the pressure from NGOs and the public to protect natural forests from plantation conversions in the face of climate change (Aggestam et al. 2020). Nevertheless, citizens in the southeast US tend to support the local forest industry and oppose too strict involvements by the government in private forest owners’ management decisions (Kreye et al. 2019). In addition, increased pellet production may help counteract other major threats to southeast forests, namely, urbanisation and expansion of agricultural areas. Furthermore, pellet mills can offer an outlet for excess logging residues and low-quality wood, which would otherwise be left on the ground to decompose or burned on-site, releasing CO_2_ to the atmosphere (Dale et al. 2017).

Within the EU, bioenergy is generally perceived to be highly supported by the public, which in turn stimulates the expansion and promotion of bioenergy policies and subsequently international trade (Ejelöv and Nilsson 2020; Magar et al. 2011). However, the confusion about different carbon accounting practices and the ‘carbon debt’ debate has shed doubt on the sustainability of the pellet trade (Parish et al. 2018) and wood-based bioenergy more generally (Searchinger et al. 2018), which may swing the pendulum of the publics’ perception against increased bioenergy use (Vainio et al. 2018). Whether the use of imported pellets from the US results in net GHG savings in the EU is contested, and depends on different modelling assumptions. Over longer timescales and with a landscape approach, lifecycle GHG emission reductions are generally assumed (Dwivedi et al. 2019; Galik and Abt 2016; Jonker et al. 2014). The reinforcing public support loop (R7) in Fig. 9 illustrates the boosting effect of public support for bioenergy on political support for bioenergy, which incentivises technological innovation in the field, potentially finding solutions to sustainability issues of bioenergy production, further increasing public support. If public support dwindles, however, the feedback effect dampens research in the bioenergy field, which leaves sustainability issues unsolved.

#### The natural environment

Changes to temperatures, precipitation patterns, frequency and intensity of extreme events or occurrence of wildfires could alter growing conditions of forests and plantations, such as soil properties or the extent of the growing season. This in turn would affect forest health, growth and productivity, regeneration success and forest structure (Halofsky et al. 2018). Damaged or stressed trees are less able to withstand e.g. strong winds or pathogen infestations (McNichol et al. 2019), exacerbating the vulnerability of the forest to climate change impacts.

**Fig. 10:**
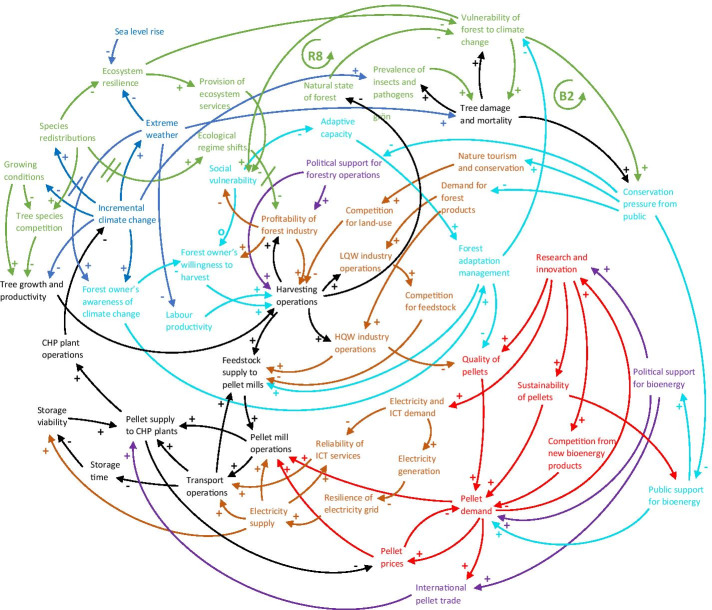
CLD showing how the sub-systems of the core supply chain (in black), other sectors and infrastructure (in light brown), the global bioenergy market (in red), policies and regulatory frameworks (in purple), and the human and social environment (in turquoise) are connected to the natural environment (in green). More connections mean greater potential for climate change impacts (in blue) to cascade through the network. R8 refers to the ecosystem services loop and B2 to the forest transformation loop described in the text. Some connections identified in the previous CLDs, but not directly related to the new connections, have been removed to improve readability. LQW = low-quality wood, HQW = high-quality wood. A description of every node and link can be found in Table 8 in the Appendix

Geographically, the southeast US is a climatic transition zone between temperate and tropical climates (Carter et al. 2018), and ecosystems are in many places located at thresholds where even relatively small climatic changes can trigger ecological regime shifts. Especially reductions in winter air temperature extremes will likely have a significant impact on species distributions, with implications for both forest tree species competition and the prevalence of tree-damaging insects and pathogens (Carter et al. 2018; Halofsky et al. 2018). The southern pine beetle already causes major economic damage to pine plantations, and increased winter temperatures may further increase over-wintering survival rates and prolong the breeding season of the insect (McNulty et al. 2013). A westward migration of tree species due to changing precipitation patterns has been noticed in the region (Fei et al. 2017), and a transformation of forests into more open woodlands due to hotter and drier conditions in the southeast is considered one of the most profound potential impacts of climate change on the US (McNulty et al. 2013). Such changes to species distributions and compositions could affect the viability of the forest industry in the region in the long term (Lal et al. 2011). The balancing forest transformation loop (B2) in Fig. 10 illustrates how climate change induced species redistributions and ecological regime shifts negatively affect the profitability of the forest industry, which causes a reduction in harvesting operations. This results in forests slowly reverting to a more natural state, which is generally believed to improve the resilience of forests to climate change. If forests are perceived to be in a resilient state, pressure from the public to protect forests decreases and political support for logging operations are free to ensue, increasing harvesting operations once more.

Many of the ecosystem services that southeast communities depend on could degrade because of climate change, which would further increase social vulnerability in the area (Carter et al. 2018). This is illustrated in Fig. 10 by the reinforcing ecosystem services loop (R8), which shows how climate change adversely affects ecosystem resilience and therefore the provision of ecosystem services, exacerbating social vulnerability. This in turn decreases the adaptive capacity of the community, resulting in less forest adaptation management measures being implemented, hence increasing the vulnerability of forests to climate change. This in turn causes social vulnerability to increase even further.

### Summary of results

The most important direct climate change impacts on the wood pellet supply chain, as well as potential cascading impacts stemming from the other sub-systems, are presented in Table 1. Direct impacts on plantations, production, storage and transportation can affect the supply of pellets in the short-term, due to e.g. extreme weather events, or in the long-term if growing conditions become less favourable in the southeast US. Furthermore, framing the wood pellet supply system in a wider socio-economic-environmental context shows that the availability of raw material for pellet production is affected by competing uses, while trends on the global bioenergy market may cause price volatility or fluctuations in demand. Policies pursuing more stringent climate and energy targets may push for an increase in the use of bioenergy, while sustainability and quality requirements may complicate the procurement of raw material.

**Table 1:** Summary of the most important direct and cascading impacts on the wood pellet supply chain. *SLR* sea level rise

Sub-system	Climate change impacts
The supply chain	Tree damage/mortality due to high temperatures, droughts, wildfires Logging and harvesting impeded by wildfires and extreme weather Pellet mills at risk from wildfires, flooding, hurricanes Storage is more challenging due to high temperatures and moist air, and facilities are at risk from wildfires, flooding, SLR, hurricanes Transportation is vulnerable to SLR, hurricanes, storm surges, flooding
Other sectors and infrastructure	Increased competition for scarce resources Decreased forestry activity reduces feedstock availability Increased demand from agriculture/tourism/nature conservation may redirect forest use towards agricultural expansion/recreation/protection Electricity outages or ICT disruptions impede mill/port operations and transport Increased competition for electricity and ICT services
Global bioenergy market	Increased prices due to adverse impacts on feedstock supply Increased or decreased supply depending on the impacts on the timber market Changes to demand because of lower quality feedstock Competition from other forms of biomass Increased demand due to technological innovations
Policies and regulatory frameworks	Increased demand due to ramping up of climate targets Changes to BMPs because of climate change induced/anticipated damages Reduced supply from small-scale forestry due to stricter sustainability criteria and uncertain political milieu
Human and social environment	Increased or decreased supply depending on forest owners’ decisions to harvest and engage in adaptation and mitigation measures Social vulnerability impedes adaptation and exacerbates climate change impacts Reduced labour productivity/availability due to climate change induced health issues Increased opposition to pine plantations, harvesting or bioenergy production by NGOs and citizens
Natural environment	Reduced growing conditions Increased prevalence of insects and pathogens Changes to species distributions and competitions Transformation of forests into open woodland could adversely affect the forest industry Ecosystem disturbances may exacerbate vulnerability and degrade ecosystem services

The availability of raw material depends on forest owners’ decision to harvest, which is conditioned on their economic situation and social context. Increased awareness of climate change among both forest owners and the public may result in increased support for bioenergy, while recognition of sustainability issues concerning carbon neutrality or adverse impacts on biodiversity may have the opposite effect. These complex linkages within the social system are further complicated by the connections to the natural environment, where climate change can reinforce existing trends, negatively affecting growing conditions, tree species distributions or the prevalence of pests and pathogens.

The CLD approach allows for an examination of potential future trajectories of change based on whether the effects of the feedback loops are reinforcing (indicating a highly dynamic situation) or balancing (indicating a more stable situation). The analysis reveals a large number of connections and feedback loops, of which only a handful received further scrutiny (see Table 2). The feedback loops described here were chosen as plausible examples of feedback effects within each of the sub-systems. An interesting observation is the fact that most of the loops in the system are reinforcing as opposed to balancing, which indicates a system in disequilibrium and may point to a tendency for amplification of climate change impacts on the system.

**Table 2:** Summary of the feedback loops selected as examples for each of the sub-systems. Note that this is not an exhaustive list of all the potential feedback loops in the system

Name	No	Loop type	Description
Supply chain loop	R1	Reinforcing	Starts with any climate change impact that affects a node in the supply chain, ultimately causing a reduction in pellet-based production of electricity and heat, requiring a ramp-up of fossil fuel-based CHP production, thus exacerbating climate change
Forest industry loop	R2	Reinforcing	Climate change increases the demand for forest products (which are considered more sustainable than e.g. plastic or steel products), which increases the profitability and hence the operations of both low-quality and high-quality wood industries. This in turn incentivises streamlining and ramping up of harvesting operations, which further increases the profitability of the forest industries
Pellet demand loop	R3	Reinforcing	Increasing pellet demand enhances pellet mill operations and causes pellet supply to increase, resulting in a pellet price drop, which further increases demand
Pellet price loop	B1	Balancing	Low pellet prices negatively affect the profitability of pellet mill operations, thus reducing pellet production and supply, causing pellet prices to increase once again
Innovation loop	R4	Reinforcing	Research and innovation increases both the quality and the sustainability of pellets, thus increasing pellet demand, which further incentivises research and innovation in the field of wood pellet production and supply
Protectionist loop	R5	Reinforcing	Adverse climate change impacts on pellet feedstock and hence pellet production in the US may result in national protectionist measures, which would hamper the international pellet trade, reducing pellet supply to European CHP plants, incurring a pellet price spike, resulting in reduced pellet demand, and thus affecting pellet production globally
Social vulnerability loop	R6	Reinforcing	Increased awareness of climate change may decrease some forest owners’ willingness to engage in harvesting operations, which negatively affects the profitability of the forest industry, thus increasing social vulnerability of the affected community as unemployment rates increase. This negatively affects the other forest owners, who become inclined to switch to more profitable activities, further reducing the profitability of the forest industry
Public support loop	R7	Reinforcing	Increased public support for bioenergy, also increases political support for bioenergy and hence incentivises research and innovation in the bioenergy field, which may solve sustainability issues of bioenergy, thus increasing public support for bioenergy even further
Ecosystem services loop	R8	Reinforcing	Climate change adversely affects ecosystem resilience and therefore the provision of ecosystem services, exacerbating social vulnerability. This in turn decreases the adaptive capacity of the community, resulting in less forest adaptation management measures being implemented, hence increasing the vulnerability of forests to climate change, which causes social vulnerability to increase even further
Forest transformation loop	B2	Balancing	Climate change induced species redistributions and ecological regime shifts negatively affect the profitability of the forest industry, causing a reduction in harvesting operations. This results in forests reverting to a natural and more resilient state, which decreases the pressure from the public to protect forests, and hence political support for logging operations are free to ensue, increasing harvesting operations once more

Taking a closer look at the complex network of connections in Fig. 10, which incorporates all the sub-systems, reveals many counteracting forces in the system as well as several links and potential cascading impacts that are not perhaps obvious but still highly relevant for the continuation of pellet supply to the EU (see the Appendix for explanations with references for all the nodes and links in the CLDs). For instance, an increased awareness of climate change among private forest owners in the US may have either an increasing or a decreasing effect on international pellet trade, depending on the situation: On the one hand, the forest owners may start implementing adaptation management measures in their forests, which would increase the amount of low-quality residues for pellet feedstock, thus decreasing the quality of and hence demand for US pellets, consequently dampening international trade. On the other hand, increasing adaptation management measures may also lead to reduced vulnerability of forests to climate change, which would lead to reduced pressure from the public to protect forests and simultaneously increase public support for pellet production, thus increasing the political support for bioenergy, which is the main driver of the international pellet trade. Additionally, increased climate change awareness may also lead to reduced willingness of forest owners to engage in harvesting, thus reducing harvesting operations and hence the profitability of the forest industry. This may increase social vulnerability in the affected communities, which decreases adaptive capacity and hence reduces adaptation management measures implemented by land owners, resulting in increased vulnerability of the natural environment to climate change, increased conservation pressure from the public and decreased political support for bioenergy and hence international pellet trade. Tangling out all of the interconnections and the potential cascading impacts they may entail for the pellet supply system would be an interesting task that is beyond the scope of this paper.

## Discussion

The study shows how a seemingly straightforward bioenergy supply chain is in fact nested within a highly complex and interconnected network of social, environmental, political and economic sub-systems, predisposing the supply chain to various cascading impacts of climate change that traverse different domains and are compounded or diminished by internal and external factors. The tendency for feedback effects in the system to be reinforcing rather than balancing, as highlighted by the CLD analysis, indicates that the system is likely to be highly dynamic in terms of future trajectories of change.

As is evident from the progressively more complicated CLDs, incorporating all the sub-systems from the analytical framework leads to an abundance of connections and causal loops. While the results give an early indication of the systemic connections and potential cascading impacts and risks, no framework, diagram or model can completely capture all the connections, causal links and effects in a complex system, and hence, this representation is inevitably a rough simplification of the supply network and its vulnerabilities. Nevertheless, it does give an indication of the importance of better understanding cascading impacts in a globally connected energy system, and how not accounting for the interdependencies within a system may exacerbate the risk of maladaptation and the implementation of policies that are not fit for purpose.

There are two main issues that may bring further uncertainties to the results. First, scenarios concerning bioenergy demand, production and supply potential and resource availability vary tremendously between studies due to e.g. differences in objectives, timeframe, resource type, studied potentials (e.g. technical, economic or theoretical), approach and methodology, and underlying assumptions of e.g. sustainability, economic and technological development and population growth (Batidzirai et al. 2012). Börjesson et al. (2017) show that scenarios assuming substantial energy efficiency improvements and high electrification rates in all sectors produce substantially lower values for bioenergy demand than scenarios with slower efficiency improvements and more limited electrification. Slade et al. (2014) highlight shortcomings and limitations with global biomass potential scenarios and conclude that these are bound with uncertainties and idealistic assumptions. Therefore, the assumption made here of increasing bioenergy utilisation is uncertain.

Second, there are issues related to modelling and data. For example, modelling of bioenergy crop production to determine availability needs to be developed (Surendran Nair et al. 2012). Nguyen and Tenhunen (2013) criticise energy crop production simulation models for not properly including socio-economic factors or local climate change impacts in their assumptions on future yields and production ranges. Similarly, data collection on the current state of bioenergy utilisation and trade within countries or regions is hampered by the insufficient detail on the origin of biomass resources, and unregistered and cascaded uses of biomass. Additionally, direct and indirect trade is insufficiently covered in statistics (Dafnomilis et al. 2017). As a result, the assumption that the southeast US will remain a major hub for pellet production and the main trade partner to the EU is debatable.

In addition, surprising events or wild cards may have unforeseen effects on interconnected systems, suddenly and surprisingly changing the status quo. For instance, the Trump administration in the US shook the climate policy arena to its core by withdrawing support for the Paris Agreement and stepping down from the climate leadership role. As previously mentioned, retracting the US from the Paris Agreement would have resulted in difficulties for many private forest owners to show compliance with EU pellet sustainability criteria, perhaps forcing the EU to look for other trade partners (Webster 2019). Similarly, the COVID-19 pandemic brought the whole world to a standstill and caused permanent changes to the economic, political and social domains. How the pandemic interacted with climate change impacts and what implications it will have for future trade or political relations important for bioenergy supply systems is yet to be seen (van den Hurk et al. 2020).

The results of this study are important for actors, organisations and governments that are engaged in any part of a bioenergy supply system. Despite a recognition among researchers and experts of the importance of using systems thinking within organisational management (Williams et al. 2017), cascading risks, failures or impacts within international supply systems are rarely acknowledged in management or adaptation strategies of companies (Goldstein et al. 2018; Tenggren et al. 2019). Additionally, cascading or cross-border impacts are rarely accounted for in national adaptation strategies or plans (Groundstroem and Juhola 2018). Different types of system-based analysis tools, such as the one highlighted here, are considered to become ever more important in supporting adaptation action in the future (Lawrence et al. 2020).

## Conclusions

A climate change assessment of bioenergy supply systems is a perfect example of a complex problem. The supply chain is embedded in a network of economic, political, social and environmental sub-systems, all of which are affected by climate change. Due to the uncertainties as to how the global bioenergy market, the political arena and human behaviour will develop in the future and how climate change impacts will affect them, there is a pressing need to further develop analyses and methodological approaches to capture some of these impacts and potential feedback loops.

This study presents an analysis of cascading impacts on the wood pellet supply chain between the US and the EU. How future demand and supply of bioenergy will be affected by EU policies is still uncertain, and therefore, this study is merely an example of one of many potential biomass supply systems that the EU may rely upon in the future. The development of pellet imports into the EU depends on many factors, such as the evolvement of sustainability criteria, increased local demand in the US, pellet price fluctuations and developments, fossil fuel price developments and the emergence of new low-carbon technologies (Mandley et al. 2020). Nevertheless, the wood pellet supply system reflects an already established supply network, which is projected to become even more important in the future, and as such is well suited for the purpose of this study.

In addition to advancing and improving methodologies to assess cascading impacts, it will become more pertinent to develop policies regarding adaptation to these risks. Even though supply chain management is the responsibility of companies, incorporation of climate risks and adaptation, especially concerning indirect, cross-border and cascading impacts, is insufficient among private companies for a variety of reasons (Goldstein et al. 2018; Tenggren et al. 2019). Hence, national governments should be involved in setting up regulatory frameworks or cooperation strategies for strengthening adaptation to such risks within companies that provide commodities or services of national importance, such as bioenergy. Identifying and planning for potential cascading impacts stemming from international bioenergy supply networks, should be a top priority for governments promoting national bioeconomies.

## Appendix

Description with references of the links between variables in the CLDs

**Table 3:** Description of the unidirectional links in the core supply chain CLD

Link	Explanation	Reference
CO_2_ level → + incremental climate change	Temperatures increase and precipitation patterns change with increasing CO_2_ levels	IPCC 2014
CO_2_ level → + tree growth and productivity	The fertilization effect increases tree growth and productivity	Susaeta et al. 2014
Incremental climate change →—tree growth and productivity	Increasing temperatures and altered precipitation patterns cause reduced tree growth and productivity in the US SE	Carter et al. 2018
Incremental climate change → + extreme weather	Increasing temperatures and reduced precipitation causes droughts and wildfires in the US SE	Carter et al. 2018
Extreme weather → + tree damage and mortality	Droughts and wildfires in the US SE cause tree damage and mortality	Carter et al. 2018
Tree damage and mortality → + harvesting operations	Increased amount of damaged or dead trees increases harvesting of wood	Barrette et al. 2015 Susaeta et al. 2014
Tree damage and mortality →—harvesting operations	In the long run, an increased tree mortality rate would reduce the amount of wood in the forest	Barrette et al. 2015 Susaeta et al. 2014
Extreme weather →—harvesting operations	Extreme weather such as storms, heavy rainfall, flooding or wildfires may delay or hinder harvesting operations	Acuna and Strandgard 2017
Tree growth and productivity → + harvesting operations	Reduced tree growth and productivity would also reduce the amount of feedstock available for harvesting	Susaeta et al. 2014
Harvesting operations → + feedstock supply to pellet mills	If harvesting operations are compromised, feedstock supply to pellet mills is reduced	Susaeta et al. 2014
Feedstock supply to pellet mills → + pellet mill operations	If feedstock supply to pellet mills decrease, pellet mill operations are reduced	Susaeta et al. 2014
Pellet mill operations → + harvesting operations	If pellet mills operations are reduced, incentives for harvesting operations are reduced	Sikkema et al. 2021
Extreme weather →—pellet mill operations	Extreme weather events, such as wildfires or flooding could damage pellet mills and hence reduce operations	Carter et al. 2018
Pellet mill operations → + transport operations	If pellet mill operations decrease, incentives to maintain transportation infrastructure in connection to mills are decreased	Awudu and Zhang 2012
Extreme weather →—transport operations	Extreme weather events are damaging for transport operations	Becker et al. 2018 Jaroszweski et al. 2010 Koetse and Rietveld 2009
Transport operations → + feedstock supply to pellet mills	If transportation is disrupted, feedstock supply to pellet mills may be interrupted or delayed	Jaroszweski et al. 2010 Koetse and Rietveld 2009
Pellet mill operations → + pellet supply to CHP plants	If pellet mill operations decrease, pellet supply to CHP plants decrease	Awudu and Zhang 2012
Transport operations → + pellet supply to CHP plants	If transportation is disrupted, pellet supply to CHP plants may be interrupted or delayed	Jaroszweski et al. 2010 Koetse and Rietveld 2009
Transport operations →—storage time	If transport operations are disrupted, pellets may have to be stored for longer periods of time	Jaroszweski et al. 2010 Koetse and Rietveld 2009
Extreme weather → + storage time	Extreme weather events such as heavy rain prevent handling of pellets, thus increasing storage time	Dafnomilis et al. 2018
Storage time →—storage viability	The longer the storage period, the higher the risk of spoiled pellets	Dafnomilis et al. 2018
Incremental climate change → + SLR	Sea levels rise with increasing climate change	IPCC 2014
SLR →—transport operations	If sea levels rise, coastal transportation may be adversely affected	Jaroszweski et al. 2010 Koetse and Rietveld 2009
SLR →—storage viability	If sea levels rise, storage facilities at ports may be damaged	Carter et al. 2018
Incremental climate change →—storage viability	A warmer and wetter climate causes challenges to storage, such as biological degradation, self-heating and ignition of pellets, dust explosions or pest infestations	Dafnomilis et al. 2018
Storage viability → + pellet supply to CHP plants	If pellets are damaged during storage, less pellets are available for CHP plants	Diaz-Chavez et al. 2019
Pellet supply to CHP plants → + CHP plant operations	When pellet supply to CHP plants is disrupted, CHP plant operations are compromised	Awudu and Zhang 2012
CHP plant operations →—incremental climate change	If the output from pellet-based CHP plant operations decrease, fossil based energy production is likely ramped up, exacerbating climate change	Sikkema et al. 2021
CHP plant operations →—CO_2_ level	If the output from pellet-based CHP plant operations decrease, fossil based energy production is likely ramped up, increasing the CO_2_ level in the atmosphere	Sikkema et al. 2021

**Table 4:** Description of the unidirectional links stemming from other sectors and infrastructure

Link	Explanation	Reference
Incremental climate change →—agriculture in the MW	Climate change is projected to make the MW less suitable for agriculture	McNulty et al. 2015
Agriculture in the MW →—agriculture in the SE	Reduced agricultural suitability in the MW has spurred an interest in agricultural expansion in the SE	McNulty et al. 2015
Agriculture in the SE → + competition for land use	Increased interest in agriculture increases competition for land	McNulty et al. 2015
Incremental climate change → + nature tourism and conservation	Nature tourism and the need for nature conservation are likely to increase with increasing climate change	Lal et al. 2011McNulty et al. 2015
Nature tourism and conservation → + competition for land use	Increased allocation of forests to tourism or conservation needs reduces the land available for plantations	Lal et al. 2011McNulty et al. 2015
Competition for land use →—harvesting operations	More competition for land use means less plantations and reduced harvesting	Lal et al. 2011McNulty et al. 2015
Harvesting operations → + profitability of forest industry	If harvesting operations are compromised, the profitability of the forest industry is reduced	Susaeta et al. 2014Lal et al. 2011
Profitability of forest industry → + harvesting operations	Reduced profitability of the forest industry reduces incentives for wood harvesting	Susaeta et al. 2014
Harvesting operations → + LQW industry operations	Reduced harvesting of wood reduces wood supply to LQW industries	Susaeta et al. 2014
LQW industry operations → + harvesting operations	Increased demand from LQW industries, increases harvesting operations	Susaeta et al. 2014
Harvesting operations → + HQW industry operations	Reduced harvesting of wood reduces wood supply to HQW industries	Lal et al. 2011
HQW industry operations → + harvesting operations	Increased demand from HQW industries, increases harvesting operations	Susaeta et al. 2014
Profitability of forest industry → + LQW industry operations	If the profitability of forest industry decreases, LQW industry operations decreases	Susaeta et al. 2014
Profitability of forest industry → + HQW industry operations	If the profitability of forest industry decreases, LQW industry operations decreases	Susaeta et al. 2014
LQW industry operations → + competition for feedstock	If LQW industry operations are reduced, competition for feedstock for pellet mills is reduced	Susaeta et al. 2014
Incremental climate change → + demand for forest products	Increased climate change increases the demand for sustainable products, such as wood products	Susaeta et al. 2014
Demand for forest products → + profitability of forest industry	Increased demand for forest products increases the profitability of the forest industry	Susaeta et al. 2014
Demand for forest products → + HQW industry operations	Increased demand for forest products increases HQW industry operations	Susaeta et al. 2014
Demand for forest products → + LQW industry operations	Increased demand for forest products increases LQW industry operations	Susaeta et al. 2014
Competition for feedstock →—feedstock supply to pellet mills	If competition for feedstock is reduced, more feedstock is available for pellet mills	Susaeta et al. 2014
HQW industry operations → + feedstock supply to pellet mills	If HQW operations are reduced, feedstock supply to pellet mills decrease	Lal et al. 2011
Incremental climate change → + electricity and ICT demand	Increased climate change increases the demand for electricity as fossil fuels are phased out	Fant et al. 2020Steinberg et al. 2020
Pellet mill operations → + electricity and ICT demand	If pellet mill operations increase, demand for electricity also increases	Uasuf and Becker 2011
Electricity and ICT demand → + electricity gen	Increased electricity demand increases electricity generation	Fant et al. 2020Steinberg et al. 2020
Electricity and ICT demand →—reliability of ICT services	Increased ICT demand put pressure on ICT infrastructure	Horrocks et al. 2010
Extreme weather →—electricity gen	Extreme weather events could damage electricity generation infrastructure	Fant et al. 2020
Extreme weather →—reliability of ICT services	Extreme weather events could disrupt ICT infrastructure	Horrocks et al. 2010
Electricity gen. →—resilience of electricity grid	Increased electricity gen. puts pressure on the electricity grid	Fant et al. 2020
Electricity gen. → + electricity supply	Increased electricity gen. leads to more electricity supply potential	Fant et al. 2020Steinberg et al. 2020
Resilience of electricity grid → + electricity supply	Grid failure or damage lead to reduced electricity supply	Fant et al. 2020Steinberg et al. 2020
Extreme weather →—electricity supply	Extreme weather events could damage electricity distribution and transmission infrastructure	Fant et al. 2020
Electricity supply → + reliability of ICT services	ICT services are dependent on an uninterrupted supply of electricity	Horrocks et al. 2010
Electricity supply → + pellet mill operations	Pellet mill operations are dependent on an uninterrupted supply of electricity	Uasuf and Becker 2011
Electricity supply → + transport operations	Interruptions to electricity supply disrupts transport operations as e.g. road lighting and traffic signals are shut down	Fant et al. 2020Markolf et al. 2019
Reliability of ICT services → + transport operations	Transportation networks are dependent on ICT services	Markolf et al. 2019
Electricity supply → + storage viability	Keeping storage conditions optimal require electricity	Uasuf and Becker 2011

**Table 5:** Description of the unidirectional links stemming from the global bioenergy market

Link	Explanation	Reference
Incremental climate change → + research and innovation	Increasing climate change increases the need for sustainable energy solutions	Mandley et al. 2020
Research and innovation → + quality of pellets	New innovations are likely to improve the quality of pellets in the future	Mandley et al. 2020
Research and innovation → + electricity and ICT demand	Technological innovations tend to rely on ICT services and electricity for energy needs	Markolf et al. 2019
Research and innovation → + sustainability of pellets	Innovations such as BECCS increases the sustainability of pellets	Fridahl and Lehtveer 2018
Research and innovation → + competition from new bioenergy products	Innovations such as pyrolysis oil increases the competition from other bioenergy products	Mandley et al. 2020
Quality of pellets → + pellet demand	Increased quality of pellets increases demand	Olsson and Hillring 2014
HQW industry operations →—quality of pellets	If demand for HQW products increase, pellet production would be mainly based on LQW, this decreasing quality	Diaz-Chavez et al. 2019Olsson and Hillring 2014
Sustainability of pellets → + pellet demand	Increased sustainability of pellets could increase demand if the ‘carbon debt’ problem would be solved	Fridahl and Lehtveer 2018
Competition from new bioenergy products →—pellet demand	If new and improved bioenergy products enter the market, demand for pellets would decrease	Mandley et al. 2020
Incremental climate change → + pellet demand	Increased climate change increases the demand for bioenergy, including wood pellets	Mandley et al. 2020
Pellet demand → + research and innovation	Increased pellet demand increases research and innovation in the field	Mandley et al. 2020
Pellet demand → + quality of pellets	Increased pellet demand may redirect higher quality wood to pellet production, hence improving the quality of pellets	Diaz-Chavez et al. 2019
Pellet demand → + pellet mill operations	If pellet demand increases, pellet mill operations are ramped up	Langholtz et al. 2014
Pellet demand → + pellet prices	If pellet demand increases, prices increase	Langholtz et al. 2014
Pellet prices →—pellet demand	If the price of pellets increase, demand decrease	Langholtz et al. 2014
Pellet prices → + pellet mill operations	If the price of pellets increases, the profitability of pellet production increases and therefore pellet mill operations are increased	Langholtz et al. 2014
Pellet prices →—CHP plant operations	If the price of pellets increase, CHP plant operations are reduced as profitability decrease	Langholtz et al. 2014
Pellet supply to CHP plants →—pellet prices	If pellet supply is reduced due to adverse impacts on feedstock production, pellet prices will increase	Langholtz et al. 2014

**Table 6:** Description of the unidirectional links stemming from policies and regulatory frameworks

Link	Explanation	Reference
Incremental climate change → + mitigation policies	Increased climate change leads to a ramp-up of mitigation policies	Mandley et al. 2020
Mitigation policies → o political support for forestry operations	Whether an increased focus on mitigation leads to political support for forestry operations depends on whether natural forests or forest management are considered more or less beneficial for mitigation	Webster 2019
Political support for forestry operations → + harvesting operations	Political support for forestry operations promotes harvesting operations	Mandley et al. 2020
Political support for forestry operations → + profitability of forest industry	Increased political support for forestry operations increases the profitability of the forest industry	Mandley et al. 2020
Mitigation policies → o BMPs promoting forestry	Mitigation policies may or may not lead to BMPs promoting forestry, depending on whether natural forests or forest management are considered more or less beneficial for mitigation	Parish et al. 2018
BMPs promoting forestry → + harvesting operations	If BMPs promote forestry, harvesting operations are increased	Parish et al. 2018
Mitigation policies → + US support for Paris Agreement	If mitigation is a priority, the US will most likely also be a party to the Paris Agreement	Webster 2019
US support for Paris Agreement →—3^rd^ party cert. of EU sustainability criteria	If the US would withdraw from the Paris Agreement, third party certification of pellet sustainability criteria would have to increase	Webster 2019
3^rd^ party cert. of EU sustainability criteria →—profitability of small-scale forestry	If the need for third party certification increases, the profitability of small-scale forestry is reduced	Diaz-Chavez et al. 2019Poudyal et al. 2019
Profitability of small-scale forestry → + feedstock supply to pellet mills	If the profitability of small-scale forestry is reduced, feedstock supply to pellet mills is also reduced as small-scale forestry is the main provider of feedstock	Conrad et al. 2011
Mitigation policies → + political support for bioenergy	Increased focus on mitigation generally increases political support for bioenergy in the EU	Mandley et al. 2020
Political support for bioenergy → + research and innovation	Increased political support for bioenergy increases incentives for research and innovation in the bioenergy field	Mandley et al. 2020
Political support for bioenergy → + pellet demand	Increased political support for bioenergy increases demand for pellets	Mandley et al. 2020
Pellet demand → + international pellet trade	Increased pellet demand in the EU drives an increase in international trade in pellets	Parish et al. 2018
Political support for bioenergy → + international pellet trade	Political support for bioenergy promotes international trade in pellets	Parish et al. 2018
International pellet trade → + pellet supply to CHP plants	Increased international pellet trade increases the availability of pellets for CHP plants	Parish et al. 2018
Pellet mill operations →—national protectionist measures	If pellet mill operations are compromised due to e.g. climate change induced supply shortages, political decisions to redirect pellet production for national purposes only may ensue	Challinor et al. 2018
National protectionist measures →—international pellet trade	National protectionist measures hinders international trade in pellets	Challinor et al. 2018

**Table 7:** Description of the unidirectional links stemming from the human and social environment

Link	Explanation	Reference
Incremental climate change → + forest owners’ awareness of climate change	Increased climate change is likely to increase awareness of climate change among forest owners due to more visible impacts	Boby et al. 2016
Extreme weather → + forest owners’ awareness of climate change	Increased occurrence of extreme weather events increases climate change awareness among forest owners	Boby et al. 2016
Forest owners’ awareness of climate change →—forest owners’ willingness to harvest	Forest owners with a strong climate change awareness tend to be less willing to engage in harvesting and instead promote regeneration of natural forests to mitigate climate change	Khanal et al. 2017
Forest owners’ awareness of climate change → + forest adaptation management	Increased climate change awareness among forest owners increases forest adaptation management measures	Khanal et al. 2017
Forest owners’ awareness of climate change → o feedstock supply to pellet mills	Increased climate change awareness among forest owners may incentivise selling of feedstock to pellet mills if forest owners believe bioenergy to be a viable mitigation strategy	Dulys-Nusbaum et al. 2019Gruchy et al. 2012
Forest owners’ willingness to harvest → + harvesting operations	Harvesting operations are dependent on the willingness of forest owners to engage in harvesting	Dulys-Nusbaum et al. 2019Gruchy et al. 2012
Profitability of forest industry → + forest owners’ willingness to harvest	Forest owners’ willingness to engage in harvesting increases with increased profitability of forestry operations	Duden et al. 2017Dulys-Nusbaum et al. 2019Gruchy et al. 2012
Extreme weather →—labour productivity	Extreme weather, such as heatwaves, reduces labour productivity	EPA 2017
Labour productivity → + harvesting operations	If labour productivity is reduced, harvesting and other forestry operations are also reduced	EPA 2017
Labour productivity → + pellet mill operations	Reduced labour productivity negatively affects pellet mill operations	EPA 2017
Labour productivity → + transport operations	Reduced labour productivity negatively affects transport operations	EPA 2017
Labour productivity →—social vulnerability	Reduced labour productivity increases social vulnerability as unemployment increases and wages decrease	Hsiang et al. 2017
Profitability of forest industry →—social vulnerability	Increased profitability of the forestry sector decreases social vulnerability in local communities due to increased employment rates and higher wages	Lal et al. 2011
Social vulnerability → o forest owners’ willingness to harvest	If social vulnerability increases among private forest owners, due to adverse economic impacts on the community, forest owners are inclined to engage in whatever activity is most profitable, be it harvesting or e.g. agriculture	Duden et al. 2017Lal et al. 2011
Social vulnerability →—adaptive capacity	Adaptive capacity decreases with increased social vulnerability	Gaither et al. 2011
Adaptive capacity → + forest adaptation management	If adaptive capacity decreases, forest adaptation management measures are less likely to be implemented	Gaither et al. 2011
Profitability of forest industry → + forest adaptation management	If the profitability of the forest industry increases, private forest owners are more likely to implement forest adaptation management measures to improve the resilience of the forest to climate change	Duden et al. 2017
Forest adaptation management → + feedstock supply to pellet mills	Forest adaptation management measures increases the availability of feedstock such as harvesting and thinning residues, which may be supplied to pellet mills	Fingerman et al. 2019
Forest adaptation management →—quality of pellets	Increased availability of residues from adaptation management increases low-quality feedstock to pellet mills	Fingerman et al. 2019
Tree damage and mortality → + conservation pressure from public	Increased adverse climate change impacts on forests may increase the pressure from the public to keep forests intact	Kreye et al. 2019Vainio et al. 2018
Conservation pressure from public →—political support for forestry operations	If public opinion favours conservation of natural forests, political support for forestry operations will decrease due to pressure from the public	Ejelöv and Nilsson 2020
Conservation pressure from public → + nature tourism and conservation	Increased pressure from the public to keep forests intact is likely to lead to increased protection of forests	Ejelöv and Nilsson 2020
Conservation pressure from public →—demand for forest products	If public opinion favours protection of natural forests, support and hence demand for forest products will likely decrease	Ejelöv and Nilsson 2020Vainio et al. 2018
Conservation pressure from public →—public support for bioenergy	If public opinion favours protection of natural forests, support for wood-based bioenergy will decrease	Vainio et al. 2018
Sustainability of pellets → + public support for bioenergy	Public support for bioenergy will increase if the sustainability of pellets increase	Vainio et al. 2018
Public support for bioenergy → + political support for bioenergy	If public support for bioenergy increases, political support for bioenergy will also increase	Ejelöv and Nilsson 2020Vainio et al. 2018
Public support for bioenergy → + pellet demand	If public support for bioenergy increases, pellet demand will also increase	Vainio et al. 2018

**Table 8:** Description of the unidirectional links stemming from the natural environment

Link	Explanation	Reference
Incremental climate change →—growing conditions	Changes to temperatures and precipitation patterns alters the growing conditions of forests	Halofsky et al. 2018
Growing conditions →—tree species competition	If growing conditions deteriorate, competition among tree species will increase	Carter et al. 2018Halofsky et al. 2018
Growing conditions → + tree growth and productivity	If growing conditions deteriorate, tree growth and productivity also deteriorate	Halofsky et al. 2018
Incremental climate change → + species redistributions	Increased climate change causes species redistributions as they migrate to more suitable environments	Carter et al. 2018Fei et al. 2017Halofsky et al. 2018
Species redistributions → + tree species competition	Species redistributions cause increased competition among tree species	Carter et al. 2018Halofsky et al. 2018
Tree species competition →—tree growth and productivity	Increased competition among tree species reduces growth and productivity of trees	Halofsky et al. 2018
Species redistributions →—ecosystem resilience	The migration and redistribution of species adversely affects the resilience of the whole ecosystem	Halofsky et al. 2018
Species redistributions → + ecological regime shifts	Species redistributions may cause ecological regime shifts	Carter et al. 2018Halofsky et al. 2018McNulty et al. 2013
Ecological regime shifts →—profitability of forest industry	Changes to tree species compositions affects the viability of the forest industry in the long run	Lal et al. 2011
Ecosystem resilience → + provision of ecosystem services	If ecosystem resilience is reduced, provision of ecosystem services is adversely affected	Carter et al. 2018Halofsky et al. 2018
Provision of ecosystem services →—social vulnerability	If provision of ecosystem services is compromised, social vulnerability in the community increases	Carter et al. 2018
Ecosystem resilience →—vulnerability of forest to climate change	If the resilience of ecosystems is reduced, vulnerability of forests to climate change increases	Halofsky et al. 2018McNichol et al. 2019
Extreme weather →—ecosystem resilience	Increased extreme weather events causes damages to forests and hence reduce ecosystem resilience	Halofsky et al. 2018McNichol et al. 2019
SLR →—ecosystem resilience	Sea level rise is detrimental for coastal ecosystems	Carter et al. 2018
Vulnerability of forest to climate change → + social vulnerability	If vulnerability of forests to climate change increases, social vulnerability also increases due to reduced ability of forests to provide ecosystem services and employment opportunities	Carter et al. 2018
Vulnerability of forest to climate change → + tree damage and mortality	Increased vulnerability of forests to climate change results in increased tree damage and mortality	Halofsky et al. 2018McNichol et al. 2019
Tree damage and mortality → + vulnerability of forest to climate change	Increased tree damage and mortality further increases vulnerability of the forest to climate change	Halofsky et al. 2018McNichol et al. 2019
Vulnerability of forest to climate change → + conservation pressure from public	If climate change vulnerability of forests increase, pressure for conservation may increase among the public	Ejelöv and Nilsson 2020Vainio et al. 2018
Forest adaptation management →—vulnerability of forest to climate change	If forest adaptation management measures are increased, vulnerability of the forest to climate change decreases	Duden et al. 2017
Tree damage and mortality → + prevalence of insects and pathogens	Damaged or dead trees are more susceptible to insect and pathogen infestations	McNichol et al. 2019
Prevalence of insects and pathogens → + tree damage and mortality	Increased prevalence of insects and pathogens causes further damage and mortality of trees	McNichol et al. 2019
Incremental climate change → + prevalence of insects and pathogens	Climate change, especially warmer winter temperatures increases the prevalence of insects and pathogens	Carter et al. 2018Halofsky et al. 2018
Harvesting operations →—natural state of forest	If harvesting operations increase, the natural state of the forest decrease as plantations take over	Duden et al. 2017Duden et al. 2018
Natural state of forest →—vulnerability of forest to climate change	Forests in their natural state are less vulnerable to climate change than disturbed forests	Aggestam et al. 2020

## Footnotes

1 Cross-border impact is defined as an impact that transcend some form of border (e.g. national, jurisdictional or ecological) (Groundstroem and Juhola 2018), while cascading and systemic impacts are often used interchangeably.

2 The RCPs (Representative Concentration Pathways) are climate change scenarios further explained by e.g. O’Neill et al. (2016).

3 The carbon debt refers to the initial release of CO_2_ upon harvesting and combustion of wood, which is supposedly offset when growing trees sequester the same amount of CO_2_ (Mitchell et al. 2012).
